# Supplementary material for: A social image recommendation system based on deep reinforcement learning
Source: PLoS One. 2024 Apr 4;19(4):e0300059. doi: 10.1371/journal.pone.0300059 (PMC10994284; doi:10.1371/journal.pone.0300059)
Supplement: S1 Appendix — (DOCX) [file pone.0300059.s001.docx]

**Appendix A: Table of Symbols**

This appendix lists all symbols used throughout the paper along with their definitions to aid in the reader's understanding.

| Symbol | Description |
| --- | --- |
| N | Number of distinct users |
| U | Set of users, $U=\left\{ u_{1}, u_{2},\ldots,u_{N} \right\}$ |
| M | Number of images |
| I | Set of images, $I=\{I_{1},I_{2},\ldots,I_{M}\}$, |
| $\boldsymbol{I}_{\boldsymbol{i}}$ | Subset of images liked by user I, $I_{i}=\{x_{1}^{i},x_{2}^{i},\ldots,x_{k}^{i}\}$ |
| S | State space |
| A | Action space |
| R | Reward |
| P | Transition probability |
| Γ | Discount factor |
| $\boldsymbol{e}_{\boldsymbol{i}}$ | the feature vector representation of item $x^{i}$ |
| $\boldsymbol{score}_{\boldsymbol{i}}$ | Scoring function for image recommendation, ${score}_{i}=e_{i}^{T}a$ |
| $\boldsymbol{L}\left( \boldsymbol{\theta}^{\boldsymbol{Q}} \right)$ | Loss function for the critic network |
| $\boldsymbol{L}\left( \boldsymbol{\theta}^{\boldsymbol{\mu}} \right)$ | Loss function for the actor network |
| $\boldsymbol{Q}\left( \boldsymbol{s,a} \vert\boldsymbol{\theta}^{\boldsymbol{Q}} \right)$ | Q-value of state-action pair under critic network parameters |
| Y | Target value for critic network update |
| $\boldsymbol{s}_{\boldsymbol{N}}$ | Next state of the user |
| $\boldsymbol{R}_{\boldsymbol{I}}$ | Recommended item at the current step |
| $\boldsymbol{w}_{\boldsymbol{ij}}$ | Weight in the self-attention mechanism |
| $\boldsymbol{x}_{\boldsymbol{j}}$ | Image previously liked by the user |
| $\boldsymbol{\beta}$ | Coefficient vector for recommendation memory |
| $\boldsymbol{score}_{\boldsymbol{i\beta}}$ | Score for image recommendation using memory vector |
