# Supplementary material for: A social image recommendation system based on deep reinforcement learning
Source: PLoS One. 2024 Apr 4;19(4):e0300059. doi: 10.1371/journal.pone.0300059 (PMC10994284; doi:10.1371/journal.pone.0300059)
Supplement: Attachment — Submitted filename: PONE-D-23-26006_Comments.pdf [file pone.0300059.s003.pdf]

PLOS ONE

A social image recommendation system based on deep reinforcement learning

--Manuscript Draft--

**Manuscript Number:**	PONE-D-23-26006
**Article Type:**	Research Article
**Full Title:**	A social image recommendation system based on deep reinforcement learning
**Short Title:**	A social image recommendation system based on deep reinforcement learning
**Corresponding Author:**	Mohsen Ebrahimi Moghaddam, Ph.D<br>Shahid Beheshti University<br>Tehran, IRAN, ISLAMIC REPUBLIC OF
**Keywords:**	image recommendation,<br>deep reinforcement learning,<br>feature extraction,<br>user preferences
**Abstract:**	Today, due to the expansion of the Internet and social networks, people are faced with a huge amount of dynamic information. Recommender systems have emerged in order to improve the problem of the phenomenon of information abundance by analyzing the background of the user's behavior and extracting the user's interests and preferences. Most of the proposed methods for social image recommender systems since now use a non-dynamic strategy, this means they cannot adopt the method by changing user preferences. In this paper we propose a dynamic Image recommender system using deep reinforcement learning frame work by employing a set of new extracted features such as emotion, style, and personality features. In the proposed method, we overcome the change of state representation definition in reinforcement learning by introducing a new method for state representation. The experimental results show that our proposed method compared to some related works significantly improve Recall@k and Precision@k for personalized image recommendation.
**Order of Authors:**	somaye ahmadkhani, Ph.D student
	Mohsen Ebrahimi Moghaddam, Ph.D
**Additional Information:**	
**Question**	**Response**
**Financial Disclosure**<br><br>Enter a financial disclosure statement that describes the sources of funding for the work included in this submission. Review the submission guidelines for detailed requirements. View published research articles from *PLOS ONE* for specific examples.<br><br>This statement is required for submission and **will appear in the published article** if the submission is accepted. Please make sure it is accurate.	The author(s) received no specific funding for this work

Powered by Editorial Manager® and ProduXion Manager® from Aries Systems Corporation

**Unfunded studies**<br>Enter: *The author(s) received no specific funding for this work.*<br><br>**Funded studies**<br>Enter a statement with the following details:<br>• Initials of the authors who received each award<br>• Grant numbers awarded to each author<br>• The full name of each funder<br>• URL of each funder website<br>• Did the sponsors or funders play any role in the study design, data collection and analysis, decision to publish, or preparation of the manuscript?<br>• **NO** - Include this sentence at the end of your statement: *The funders had no role in study design, data collection and analysis, decision to publish, or preparation of the manuscript.*<br>• **YES** - Specify the role(s) played.<br><br>* typeset	
**Competing Interests**<br><br>Use the instructions below to enter a competing interest statement for this submission. On behalf of all authors, disclose any competing interests that could be perceived to bias this work—acknowledging all financial support and any other relevant financial or non-financial competing interests.<br><br>This statement is required for submission and **will appear in the published article** if the submission is accepted. Please make sure it is accurate and that any funding sources listed in your Funding Information later in the submission form are also declared in your Financial Disclosure statement.<br><br>View published research articles from *PLOS ONE* for specific examples.	The authors have declared that no competing interests exist.

**NO authors have competing interests**<br><br>Enter: *The authors have declared that no competing interests exist.*<br><br>**Authors with competing interests**<br><br>Enter competing interest details beginning with this statement:<br><br>*I have read the journal's policy and the authors of this manuscript have the following competing interests: [insert competing interests here]*<br><br>* typeset	
**Ethics Statement**<br><br>Enter an ethics statement for this submission. This statement is required if the study involved:<br><br>• Human participants<br>• Human specimens or tissue<br>• Vertebrate animals or cephalopods<br>• Vertebrate embryos or tissues<br>• Field research<br><br>Write "N/A" if the submission does not require an ethics statement.<br><br>General guidance is provided below. Consult the submission guidelines for detailed instructions. **Make sure that all information entered here is included in the Methods section of the manuscript.**	N/A

Powered by Editorial Manager® and ProduXion Manager® from Aries Systems Corporation

**Format for specific study types**<br><br>**Human Subject Research (involving human participants and/or tissue)**<br>• Give the name of the institutional review board or ethics committee that approved the study<br>• Include the approval number and/or a statement indicating approval of this research<br>• Indicate the form of consent obtained (written/oral) or the reason that consent was not obtained (e.g. the data were analyzed anonymously)<br><br>**Animal Research (involving vertebrate animals, embryos or tissues)**<br>• Provide the name of the Institutional Animal Care and Use Committee (IACUC) or other relevant ethics board that reviewed the study protocol, and indicate whether they approved this research or granted a formal waiver of ethical approval<br>• Include an approval number if one was obtained<br>• If the study involved *non-human primates*, add *additional details* about animal welfare and steps taken to ameliorate suffering<br>• If anesthesia, euthanasia, or any kind of animal sacrifice is part of the study, include briefly which substances and/or methods were applied<br><br>**Field Research**<br><br>Include the following details if this study involves the collection of plant, animal, or other materials from a natural setting:<br>• Field permit number<br>• Name of the institution or relevant body that granted permission	
**Data Availability**<br><br>Authors are required to make all data underlying the findings described fully available, without restriction, and from the time of publication. PLOS allows rare exceptions to address legal and ethical concerns. See the PLOS Data Policy and FAQ for detailed information.	Yes - all data are fully available without restriction

Powered by Editorial Manager® and ProduXion Manager® from Aries Systems Corporation

A Data Availability Statement describing where the data can be found is required at submission. Your answers to this question constitute the Data Availability Statement and **will be published in the article**, if accepted.<br><br>**Important:** Stating 'data available on request from the author' is not sufficient. If your data are only available upon request, select 'No' for the first question and explain your exceptional situation in the text box.<br><br>Do the authors confirm that all data underlying the findings described in their manuscript are fully available without restriction?	
**Describe where the data may be found in full sentences. If you are copying our sample text, replace any instances of XXX with the appropriate details.**<br><br>• If the data are **held or will be held in a public repository**, include URLs, accession numbers or DOIs. If this information will only be available after acceptance, indicate this by ticking the box below. For example: *All XXX files are available from the XXX database (accession number(s) XXX, XXX.).*<br>• If the data are all contained **within the manuscript and/or Supporting Information files**, enter the following: *All relevant data are within the manuscript and its Supporting Information files.*<br>• If neither of these applies but you are able to provide **details of access elsewhere**, with or without limitations, please do so. For example:<br><br>*Data cannot be shared publicly because of [XXX]. Data are available from the XXX Institutional Data Access / Ethics Committee (contact via XXX) for researchers who meet the criteria for access to confidential data.*<br><br>*The data underlying the results presented in the study are available from (include the name of the third party*	The data set underlying the results presented in the study are available from references 1, 28, 33, and 38 that are referred to in the article.

Powered by Editorial Manager® and ProduXion Manager® from Aries Systems Corporation

and contact information or URL).<br>• This text is appropriate if the data are owned by a third party and authors do not have permission to share the data.<br><br>* typeset	
Additional data availability information:	

Powered by Editorial Manager® and ProduXion Manager® from Aries Systems Corporation

Manuscript Click here to access/download;Manuscript;Article.docx

A social image recommendation system based on deep reinforcement learning

Somaye Ahmadkhani, Mohsen Ebrahimi Moghaddam

Shahid Beheshti University, Faculty of Computer Science and Engineering, Tehran, Iran

Abstract

Today, due to the expansion of the Internet and social networks, people are faced with a huge amount of dynamic information. Recommender systems have emerged in order to improve the problem of the phenomenon of informat[illegible] abundance by analyzing the background of the user's behavior and extracting the user's interests and preferences. Most of the proposed methods for social image recommender systems since now use a non-dynamic strategy, this means they cannot adopt the method by changing user preferences. In this paper we propose a dynamic Image recommender system using deep reinforcement learning frame work by employing a set of new extracted features such as emotion, style, and personality features. In the proposed method, we overcome the change of state representation definition in reinforcement learning by introducing a new method for state representation. The experimental results show that our proposed method compared to some related works significantly improve Recall@k and Precision@k for personalized image recommendation.

Keywords: image recommendation, deep reinforcement learning, feature extraction, user preferences

1 Introduction

In recent years, with development of the Internet and social networks, impressive research effort has been directed at recommender systems. Because in the face of the huge volume and variety of information, we need a system that can automatically identify and provide the user's interests. In spite of the various advances in recommender systems, existing recommender systems require further improvements to provide more efficient recommendations applicable to a broader range of applications. The exponential increase of images as an important source of information in online services and social networks prompted us to investigate the image-based recommender system of social networks. Many researches have been done in this field that focuses on handcrafted features to identify user interests [1, 2], and many studies try to use deep learning models [3-5] for representation and recommendation images. Furthermore, some similarity measures [6] and classification methods [7] have been used for recommendation in image recommender systems. The remarkable point in all this is that the recommendation is considered as a static procedure and assumes that the user’s underlying preference keeps unchanged, while the system should be in dynamic interaction with the user because the user’s preference is dynamic with respect to time. Therefore, we need a novel recommendation method that considers recommendation a dynamic process.

Today, reinforcement learning is used for its high ability to solve complex problems requiring dynamic modeling and long-term planning. The use of reinforcement learning in recommender systems is not new and has been used in old works. Model-based RL techniques such as POMDP [8] and Q-learning [9] were among the first RL techniques for modeling recommendation methods. Nevertheless, when the number of items to recommend is large, these methods could be more efficient due to time complexity. Later, model-free RL techniques were also used for recommendation into two categories: value-based [10-12] and policy-based [12-14].

Although using RL for recommender systems is not new, mainly because of scalability problems, traditional RL algorithms were not very practical. With the advent of deep reinforcement learning (DRL), a new trend emerged in this field that enabled the application of RL to recommendation problems with large state and action spaces [15].

Some of the studies use reinforcement learning for recommendation. The authors in [16] propose a recommendation framework based on deep reinforcement learning. They model the interactions between the users and recommender systems using an Actor-Critic reinforcement learning scheme and consider both dynamic adaptation and long-term rewards. Xiangyu et al. [12] used deep reinforcement learning to automatically learn optimal recommendation strategies and model the recommendation as a Markov decision process.

Due to operationality of recommender systems based on reinforcement learning for real-world problems, we propose deep Reinforcement Learning to define the sequential interactions between users and the recommender system to learn the optimal strategies from users’ feedback automatically. The proposed method consists of two main parts, and there are innovations in each part. In the first part, we propose to extract a set of different features from images that

represent the characteristics and preferences of the user. To this end, we use three new components: emotion analysis, personality recognition, and style detection to represent users and improve the social image recommender system. In the second part, we propose a new framework based on actor-critic reinforcement learning and with a state representation module to have a dynamic recommender system. Our contribution in this work can be summarized as follows:

1) Propose an image recommender system in a new deep reinforcement learning framework

2) Introducing a new method for state representation.

3) Introducing three components (style, Emotion, personality) that can be useful in images recommendation. Create user's characteristics vector and investigate its effect on the recommended images to each user.

The continuation of this paper is as follows. In Section 2 related work and background are presented. The proposed methods are introduced in Section 3. In Section 4 experimental details and results are discussed. Finally, we conclude this paper in Section 4 and discuss some future work.

2 Image recommendation related works

In this section, we divide recommender systems into two categories Non RL or traditional recommendation systems and RL based recommendation systems and review related works. Recommender systems are one of the topics of researchers' attention, which are used in different fields [17] [18]. Traditional recommender systems can be divided into three categories collaborative filtering, content-based filtering, and hybrid [18]. Collaborative filtering recommendation is based on rates given to the items by the users and using rates to find similar users. Content-based filtering is based on content features of items to find similarities between items. Hybrid methods use the capabilities of both methods. By increasing number of images on the Internet and social networks, image recommender systems became an important issue and have received attention. Therefore, we focus on image-based recommendations, especially social images.

Some studies have investigated feature extraction methods from images to understand user interests. Lovato et al. [1] considered the images tagged as favorites by a user and extracted handcrafted features from them such as HSV statistics, Use of light, and etc. Guntuku et al. [7], in addition to low-level features [1], extracted a set of high-level features such as Head and Upperbody recognition, Visual Clutter, tag, etc. They proposed a deep bimodal knowledge representation model that increased efficiency by using visual and tag features. The method presented in [19] extracts various features, applies feature selection to take the important features, and then uses fuzzy inference system for image recommendation. In [20], style is used in the recommendation and it shows that the use of style improves the result. In some previous researches, image-level features, deep methods, and social information have been noticed to determine user preferences [21-23]. However, defining a new component related to user preferences can be useful.

Most traditional recommendation methods involve user-item interaction modeling with supervised learning such as classification, memory-based content filtering from user history, etc. These methods ignore the dependence during successive time steps. Unlike the supervised learning setting, where a guide tells you the right action, to better reflect the user-system interaction, it is widely agreed that the formulation of the problem as a sequential decision problem can be better [15]. Therefore, it can be solved by reinforcement learning relies on the environment to discover the right action. In reinforcement learning, the learning process is through interaction with an environment.

Shani et al. [8] use of Markov decision processes (MDPs) for recommender systems to consider the long-term effect of recommendations and the expected value of each recommendation. Taghipour et al. [9] Use of Q-Learning to model web page recommendation. However, these model-based RL techniques [9] are inapplicable when there are many candidate items for recommendation, because updating the model requires a time-consuming dynamic programming step. Therefore, model-free RL techniques were preferred for use in recommender systems. These techniques can be divided into two categories, value-based [10, 11] and policy-based [13, 14, 19].

In value-based approaches, for a given state, one must calculate the Q values of all available actions, then the action that has the maximum Q-value is selected as the best action. Therefore, when we face a very large action space,, the approaches may become very inefficient.

The policy-based approaches generate a continuous parameter vector to represent an action [13, 14, 19]. This vector can generate the recommendation and update the Q-value evaluator; so can overcome the inefficiency drawbacks.

As deep neural networks developed, so did deep reinforcement learning. Deep RL techniques have recently attracted recommender systems attention because they enable RL use in problems with large state and action spaces. Huang et al. [24] considered the recommendation process as a Markov decision process and proposed a top-N model based on deep reinforcement learning for long-term prediction wherein recurrent neural network is used to simulate the interactions between the recommender system and user. Zheng et al. [10] applied deep Q-learning in news

recommendations to effectively model the dynamic news features and user preferences. Liu et al. [16] used actor-critic reinforcement learning to model interactions between users and recommender systems to have dynamic adaptation and long-term rewards. Zhao et al. [12] proposed List-wise recommendations based on Deep Reinforcement Learning to automatically learn the optimal recommendation strategies. In another study, Zhao et al. [25] used Deep Reinforcement Learning for page-wise recommendation to automatically learn the optimal recommendation strategies and optimizes a page of items simultaneously.

3 Proposed Method

Figure 1 shows the workflow of the proposed method that consists of two main parts, feature extraction and deep reinforcement learning to learn the recommendation process. For feature extraction, we have proposed the use of three new components consisting of emotion analysis, personality recognition, and style detection to improve the social image recommender system. In the second part, we proposed a new framework based on actor-critic reinforcement learning to have a dynamic recommender system. In the following, we will first describe the methods used to feature extraction, and then we will present the proposed recommender system based on deep reinforcement learning and the extracted features.

Fig. 1 Workflow of the proposed method

3-1) Feature Extraction

In the proposed method framework of the recommender system, there are four components of feature extraction (Figure 1). Visual features, Style feature extractor, Emotion feature extractor, and personality feature extractor (These features listed in Table 1). The model designed and used for each is described below. For each image, we have extracted a[illegible]otion vector that expresses the em[illegible]s in the image, a style vector that expresses the most prominent styles in the image, and a personality vector that expresses the prominent features of the personality in the image. We interpret this idea in such a way that the user tags an image as his favorite that matches his interests in terms of emotion, personality, and style. In order to extract these three different features, we must first train a model for each one, and then we can extract the corresponding feature vector by giving an image to each of these models. After extracting the desired components, we use reinforcement learning and propose a new social image recommender system.

A) Emotion feature extractor

Understanding emotions in images has attracted many interests due to its various applications. Many researchers inspired by psychology and artistic principles, investigated various features extracted from images to automatically obtain a single emotion for each image [26, 27]. In the past few years, with the popularity of convolutional neural networks (CNNs), researchers [28, 29] have used CNNs to recognize image sentiments and demonstrate that deep features perform better than hand-tuned features. Recent algorithms in convolutional neural networks significantly improve emotion classification, which aims to detect differences between emotion categories and assign a predominant label to each image. Some studies have been done on increasing the depth of neural networks, and in some cases, it has been shown that as the depth of the network increases, the ability of the network to extract higher-level features improves. EfficientNet neural network [30] states that CNN models should be scaled meaningfully to achieve better accuracy and efficiency. [30] states that carefully balancing the network's depth, width, and resolution can improve performance. Based on this, a new scaling method has been proposed that scales all the dimensions of depth/width/resolution uniformly using a simple, very effective combination factor.

In the present paper, utilizing of the new deep neural network, EfficientNet, has been investigated, which is expected to lead to better results. In this method, instead of expanding the network in only one of the cases of depth, width, and resolution, this expansion is done in a combined manner to have the highest efficiency with a smaller number of parameters. We have suggested using the fine-tuned EfficientNet network to analyze the emotion of the images.

B) Personality feature extractor

Personality analysis can be one of the most important methods for distinguishing between users in their preferences and behavior; since far, many research investigated personality analysis [31, 32]. In this research, we have examined personality analysis as one of the influential components in the social image recommender system. For this purpose, to predict personality traits we use pre-trained CNNs for image classification. These networks use a large set of images for training, and the intermediate layers depict the semantics of the general visual appearance. We use the power of these networks and fine-tuned them for our problem to learn visual representations correlated with personality traits. We have taken an approach similar to what was implemented in [33]. This study models the personality traits of users based on the Five Factor Theory. Therefore, five distinct binary classifications are considered for each trait. We have considered CNN networks, trained on the ImageNet, fine-tuning the network, and changed the last layer to adapt to binary classifications for each trait.

C) Style feature extractor

Image style plays an important role in how the image looks. Automatic image style recognition is crucial for many applications, including artwork analysis, photo organization, and image retrieval [34-36], however, using style in social image recommender systems has not received much attention. This study proposes image style detection as a component to understand user preferences better. For this purpose, we must first obtain the style vector for each image. Our previous work [20] proposed an image style detection method based on deep correlation features and a compact convolutional transformer (CCT). This method is based on convolution and tries to preserve local information. The idea in this method is that a new convolutional block is proposed instead of the simple convolutional block in CCT. For this purpose, we use VGG-19 pre-trained convolutional layers, which are trained using the ImageNet dataset as a convolutional block, and fine-tuned part of it during the compression transform learning process. In this paper, we have used the proposed method in [20] to extract the style features of images.

D) Visual Feature

Visual features in Table 1 are part of the features used in [1].

Table 1 List of features

	Category	Name	Feature Dimensions
**Visual Feature**		**Use of light**	1
		**HSV statistics**	3
		**E[illegible]n based**	3
		**Hue Circular Variance**	1
		**Colorfulness**	1
		**Color name**	11
		**Ent[illegible]**	1
		**Wavelet textures**	12
		**Tamura**	3
		**GCLM features**	12
		**Edges**	1
		**Level of detail**	1
		**Regions**	1
		**Low depth of field (DOF)**	3
		**Faces**	1
		**GIST descriptors**	24
**Style Feature**	Atmosphere	**Hazy**	1
		**Sunny**	1
	Color	**Bright**	1

		**Pastal**	1
	Composition	**Detailed**	1
		**Geometric**	1
		**Minimal**	1
		**Texture**	1
	Mood	**Serene**	1
		**Melancholy**	1
		**Ethereal**	1
**Emotion Feature**		**Sadness**	1
		**Fear**	1
		**Excitement**	1
		**Disgust**	1
		**Contentment**	1
		**Awe**	1
		**Anger**	1
		**Amusement**	1
**Personality Feature**		**Openness**	1
		**Conscientiousness**	1
		**Extraversion**	1
		**Neuroticism**	1
		**Agreeableness**	1

3.2) Recommendation based on Reinforcement Learning

We model our proposed method in the reinforcement learning setting. An agent is a recommendation system in our context that interacts with the environment (users) and receives rewards from the environment (feedback from users). Rewards serve as an index of whether the course of action the agent is taking is right or wrong. The agent is constantly in contact with the environment and eventually learns to take the right action through the feedback received from the environment over a period of time. The underlying reinforcement learning model is the Markov Decision Process (MDP) includes a sequence of states, actions, and rewards. MDP is determined by five componen[illegible] follows:

State space *S*: The agent's situation at any moment and the environment has created it. In our work, the status s indicates the user's positive interaction history with the recommender, and we have proposed a state representation module to represent it (section 3-2-s1).

Action space A: An action a ∈ A includes possible reactions that the agent may show when facing the current state. It can be a list of items recommended to user at current state.

Reward R: Immediate feedback sent to the agent by the environment after evaluating each action.

Transition probability P: the probability of state transition from s_t to s_{t+1} when agent performs action a_t.

Discount factor γ: It is a factor that measures the present value of long-term rewards. Its value is between 0 and 1. If γ = 0, the recommender ignores long-term rewards and considers only immediate ones. But, when γ = 1, the recommender considers long-term and immediate rewards equally important.

As aforementioned most previous work has focused only on recommendations as a static process and using pre-trained models for recommendations, and these cannot simulate the interaction process between users and their systems. Therefore, we propose a new social images recommender system in the deep reinforcement learning framework. To this end, deep deterministic policy gradient (DDPG) Algorithm has been used to provide recommendations. DDPG is an actor-critic technique that combine[illegible]h Q-learning and Policy gradients. This method consists of two models, the Actor network and the Critic network.
The actor or policy network takes the state as input and generates an action for a given user based on her state. The user's state vector is determined at each step using the state representation module. This module obtains the state of the user based on the history of images that have been liked by the user. (The proposed module for state representation is described in Section 3-2-1. The actor-network consists of two ReLU [illegible]s and one Tanh layer, and we have used a skip connection in the penultimate layer. The user state is given as input to the actor network to generate the action vector. The generated action vector is used to provide recommendations to the user. Given that there are many images

to recommend to the user, we use the scoring function based on the inner product defined as follows to determine the recommended image.

$$score_i = e_i^T a \qquad (1)$$

Where a is the output of Actor and e_i expression is the vector representation of item i. After calculating the scores, the images are ranked based on the scores, and the image with the top rank is recommended.

The critic network: The Critic is a Deep Q-Network designed as a deep neural network parameterized $Q(s, a)$. The generated action vector and the user's state are given as input to the critic network to determine how good the generated action is. According to $Q(s, a)$, the Actor network parameters are updated in order to improve the performance of action a, that is, to enhance $Q(s, a)$.

Given that there are many images to recommend to the user and we have to determine the recommended image with the help of the Action vector, in order to help the recommendation process, we have proposed considering a memory of the recommendation history. This memory keeps all the images recommended to the user, both those that are liked by the user and those that are not. We apply the Lasso algorithm to obtain the vector of Lasso coefficients on the information stored in this memory. Then use the dot product between the obtained vector of coefficients (β) and all images those are candidate for recommendation, to select a list of images, that are better candidate for recommendation.

$$score_{i\beta} = x_i \beta \qquad (2)$$

After calculate $score_{i\beta}$ for all x_i, we consider that those who generate high scores are better candidates for recommendation, and use the dot product between the list of selected images and the action vector obtained from the actor network to create recommendations using equation (1).

The reward function is defined as eq.2. When an image is recommended to the user if that image is liked by the user, a positive reward is returned to the system, and if it is not liked, a negative reward is returned to the system.

$$Reward(Image) = \begin{cases} 1 & If\ the\ user\ likes\ the\ image \\ -1 & If\ the\ user\ does\ not\ like\ the\ image \end{cases}$$

(3)

3-2-1) state representation module

State representation in reinforcement learning is a challenging problem. Therefore, we have proposed a new method for state representation. To this end, we have used the self-attention [37] idea to represent the user's state. In such a way that at every step, when an image is recommended to the user, if the user likes it, we get the self-attention of the recommended images with other images in the collection that the user likes. Equation 3 shows how to update the user state. We display the current status with s_c, the next state with s_N, and PositiveS[illegible]defines a set of recommended items in previous steps that are liked by the user.

$$s_N = \begin{cases} (R_I + selfAttentionRepresentation(R_I, PositiveState))/2 & if\ \ R_I\ Liked\ by\ user \\ mean(PositiveState) & else \end{cases} \qquad (4)$$

Where R_I define recommended item in this time.

Figure 5 shows the proposed approach to determine the next state of the user. Let x_2 be the image that the user recently liked. We assume $\{x_1, x_3, x_4\}$ is the user's favorite collection of images. The following method is used to calculate the next state of the user (s).

Self-attention is the central mechanism of the Transformer. The purpose of Self-Attention is to measure the dependencies of the components in a sequence with each other in order to have a more accurate perception of the whole sequence. In our work, it is assumed that we have a sequence of images liked by the user, and the x2 that was recently liked by the user enters the state representation module. To produce the expected output of the entered image (next state of the user), similar to self-attention, a weighted average on the inputs (here, the image that was recently liked was entered, and the liked images from the previous steps are considered as inputs) is calculated.

To generate the output vector s, we take the weighted average operation on all available input vectors:

$$s = \sum_j w_{ij} x_j \qquad (5)$$

Where j indexes over the whole sequence and the sum of the weights to one over all j, the weight w_{ij} is derived from a function over x_i and x_j. The simplest option for this function is the dot product:

$$w'_{ij} = \mathbf{x}_i{}^{\mathrm{T}} \mathbf{x}_j \,. \qquad (6)$$

Note that x_i is the input vector recently liked, and x_j is the image previously liked by the user.

The dot product gives us a value between negative infinity and positive infinity, so we use a softmax to map the values to [0,1] and make sure they sum to 1 over the entire sequence:

$$w_{ij} = \frac{\exp w'_{ij}}{\sum_j \exp w'_{ij}} \,. \qquad (7)$$

Fig. 2 State representation using self-attention

4 Experiment

4.1 Dataset

In this study, we used four datasets. 1) Flickr Style [38] for recognizing image style. The original dataset consisted of 80,000 images with style labels and was classified into 20 labels. We could not collect all the images because some were unlinked from Flickr. In this study, 15 style labels are used, and we have 2900 images for each style. Fig. 3 shows seven sample images from this dataset with serene, melancholy, ethereal, noir, vintage, romantic, and horror styles.

2) The data set introduced in [28] was used to detect the emotion of the image. This dataset contains images of eight emotions defined in Table 2, including amusement, anger, awe, contentment, disgust, excitement, fear, and sadness. Due to the loss of some images, the data set used in this article contains fewer images than the original data set. Table 2 shows eight emotions and the number of images of each emotion. Eight sample images with their emotion are shown in Fig. 4.

3) PsychoFlickr dataset is used for personality analysis [33]. A collection of 60,000 images tagged as favorites by 300 Pro Flickr users (200 randomly selected favorites per user).

4) To evaluate image recommendation, we employ part of the dataset used in [1]. We consider a collection of 4000 images belonging to 20 users from Flickr. For each user, there are 200 images tagged as favorites. Random samples of images tagged as favorites by users are shown in Fig. 5.

The first three datasets are respectively used to train the three methods of style extraction, emotion extraction, and personality analysis, and the fourth dataset is used to check the performance of the proposed recommender system.

Table 2: Statistics of the current labeled image data set.

Sadness	Fear	Excitement	Disgust	Contentment	Awe	Anger	Amusement	Sum
2577	969	2725	1592	5130	2881	1176	4724	**21774**

Fig. 3 Random sample images from the Flickr datasets. From left to right, the styles are noir, romantic, horror, and vintage in the first row. From left to right, the styles are ethereal, melancholy, and serene in the second row.

Fig. 4. Sample images of eight different categories of emotions. Top row: four positive emotions and bottom row: four negative emotions.

Fig. 5. Random samples of the dataset used for recommendation

4.2 Implementation results of feature extraction methods

We must first extract the mentioned features to provide a recommendation system based on reinforcement learning. We need to train models to extract emotion, personality, and style characteristics. In the following the implemented models and their results for extracting these components have been stated briefly.

Emotion. We first used a system to recognize the emotion of each image. We have built our framework based on the EfficientNetB1 model. First, the network is set up with the trained weights for the image classification task using the ImageNet dataset, and then we fine-tune the network for our problem. Since the class number of the effective dataset is not the same as ImageNet, the last fully connected layer is changed to the required number of classes of our target dataset, which can generate a probability distribution on the emotional labels. Due to system limitations, the size of the images has been resized to 200×200.

Table 3. The accuracy of different methods for emotion recognition

Method	Accuracy
FinetuneFM VGG19 (ImageNet)	53.49
EfficientNetB1 (ImageNet)	64.88
EfficientNetB2 (ImageNet)	64.02
FinetuneFMEfficientNetB1 (ImageNet)	**66.69**

As shown in Table 2, the dataset used for the emotion recognition model includes 21,000 images, of which 80% are used for training, 15% for testing, and 5% for validation. Table 3 reports the results obtained from the four methods implemented to recognize emotions. The results show that the FinetuneFMEfficientNetB1 (Imagenet), the Finetune of the EfficientNetB1 network leads to better results. Therefore, we improved the recommender system based on emotion analysis based on this model.

Personality. For personality feature extraction, we have taken an approach similar to what was implemented in [33]. We attempts to model the personality traits of users based on the Five Factor Theory. Therefore, five distinct binary classifications are considered for each trait. For each trait, the range o[illegible]ues is divided into three parts: Values that are below the first quartile, low set. Values above the third quartile are the upper set and the middle set. For the binary classification problem, to further distinguish between the two classes, we only select users with values in the low and high sets.

We have considered two CNNs networks, VGG16 and VGG19, trained on the ImageNet and changed the last layer to adapt to the personality recognition problem. These trained networks are ideal candidates for fine-tuning because they are trained on many images (1.2 million) and many classes (1000 object categories), providing very strong representational power. The results of implantation have been shown in Table 4. In our experiment, the size of the image has been considered 160×160. We investigate attributed traits, because in recommender system we only have image like by user and we cannot find any information about their personality.

Table 4. The accuracy of different methods for personality analysis

	**Attributed traits**				
	O	C	E	A	N
Fine-tune VGG16	57.16	60.47	61.02	57.96	62.35
Fine-tune VGG19	58.4	62.08	63.79	60.87	64.33

Style. Our previous work [20] proposed an image style detection method based on deep correlation features and a compact convolutional transformer (CCT). This method is based on convolution and tries to preserve local information. The idea in this method is that a new convolutional block is proposed instead of the simple convolutional block in CCT. For this purpose, we use VGG-19 pre-trained convolutional layers, which are trained using the ImageNet dataset as a convolutional block, and fine-tuned part of it during the compression transform learning process. In this paper, we have used this method [20] to extract the style features of images.

4.2 Implementation Results and Analysis

In this section, we investigate the effectiveness of the proposed method for social image recommendation system. For this purpose, we have reviewed related works and compared the proposed method with some representative baseline methods. The experimental results have been compared with the results obtained from the following methods:
(i) FGM, this method uses visual features and then applies FGM for simultaneous feature selec[illegible] and classification [7].
(ii) LASSO, uses visual features and then LASSO regressor [1].
(iii) DeepFeatNN, Deep Features are extracted to learn user preferences. This method generates a user feature vector using averages all the image features liked by the user and then recommends an image that is more similar to the user's vector [6].
(ix) VOFeatFGMPFIS [19], this method use Visual and Object Features. For object features, VGGnet trained on 1000 object from ImageNet tag set [39] was used.
(xi) VisualFeat.FTFMCT. FGM, This method uses style and a visual feature vector and then applies FGM for simultaneous feature selection and classification [20].

We consider a collection of 4000 images belonging to 20 users from Flickr [1] to evaluate image recommendation. For each user, there are 200 images tagged as favorites. To compare the above methods, we have divided the data set 50%/50% into test and train data. We only have the liked images of the user and we don't have the images that are not liked by the user, but we need two sets of positive and negative samples for each user. Therefore, for positive samples we selected 100 images of 200 images “liked” by the user. To determine negative samples, we calculated the similarity between users and then selected 100 images of 200 images “liked” by each of ten users with the most distance from the user. For the test set the remaining images of users are used.

In our experiments, we repeat 400 episodes for the proposed method, and 100 images are recommended to the user in each episode. In each episode, we store 100 images recommended to the user, whether he likes them or not, in memory, and we call it the recommendation history. At the beginning of each episode, we apply lasso regression on the data stored in the history of recommendations to determine the features that are most important to the user for liking the image. We use the vector obtained from Lasso to filter a large set of images that are candidates for recommendation to the user. Then we use the set of images obtained from this filter to apply equation (1) and obtain the score of each image. After calculating the scores with equation (1), the images are ranked based on the scores and the image with the highest score will be recommended. These repetitions and the use of the lasso filter help update the parameters of the actor and critic networks according to the user's interests and advance the system toward providing better recommendations. At the beginning of the system, we set the initial status with one image liked by the user, and at each step, the user's state is updated by the state representation module. This issue can solve the problem of cold start which is one of the problems of recommender systems because the system starts with the least possible information from the user and updates itself with the user's preferences at every step.

After training, for each user, the test images are ranked based on the score given by the recommendation model. We implement different methods and report the top k recommended images, where k is adjustable. We know only the relevant images (user “likes”); therefore, we use Precision@k and Recall@k to evaluate the performance of the methods. Precision@k means the proportion of relevant top k images, and Recall@k means the proportion of relevant images in the top k. The formulation of Recall@k and Precision@k is as follows:

$$\text{Recall} = \frac{\text{Relevant\_Items\_Recommended in top}\,k}{\text{Relevant\_Items}} \quad (8)$$

$$\text{Precision} = \frac{\text{Relevant\_Items\_Recommended in top k}}{\text{k\_Items\_Recommended}}$$

Fig. 6 Recall@k for different k values

Fig. 7 (a) Recall@k for different k values and Precision@k for different k values

The dimension of the image embedding vector determines based on features extracted and is 107, and we set the discounted factor $\gamma = 0.\ 5$.

We calculate the recall@K and Precision@K on all users using Eqs. (2) and (3). Fig 6 and 7 show the results, we can see that the proposed method outperformed baselines, demonstrating our proposed method's effectiveness. Recall@K is used to calculate the proportion of relevant items in the top-K. Precision@K calculates the proportion of relevant top-K items (liked by the user).

The 15 top recommended images for one of the users with different methods are shown in Fig 8. In this presentation, we have sorted the test images based on the score obtained from different methods. Then we identified 15 images with the highest score as top K recommended image by each method. 15 top image recommended to the user using proposed method can be seen in Fig 8.f. As you can see, only two of the 15 images recommended to the user were not part of the user's favorite collection. While in other methods, a larger number of recommended images are not part of the user's favorite collection, which shows the superiority of the proposed method over other methods. In fact, we have considered user preferences from different aspects and analyzed more features of the user; applying different components, emotion, style, and personality, to recognize user preferences. On the other hand, we have used an interactive method with the user, and the system is updated according to the user's preferences, so we got better results.

Fig. 8 (a) some samples of training images for user2. (b)–(f) 15 top images recommended to user2 by different methods. Incorrect recommendations are highlighted in red.

5. CONCLUSION

In this paper, we propose a new deep reinforcement learning social image recommender system to learn the optimal recommendation strategies automatically and [illegible]ide a system that can directly interact with the user and get feedback to improve and update the system strategies continuously. Also, RL base recommendations can learn a strategy that maximizes the long-term cumulative reward from users. To represent the user's state, we have proposed the state representation module, which updates the user's state in each step using the images liked by the user in the previous steps. in addition, we propose using a different collection of features, Emotion, style, and personality, to recognize the user's preferences. The results show the superiority of the proposed method over other methods. Using other behavioral features related to user behavior is possible in future works. Introduce methods that can give more accuracy in detecting emotion, style, and personality can be investigated in the future, and as a result, the recommender system is expected to improve.

References

1. Lovato, P., et al., *Faved! biometrics: Tell me which image you like and I'll tell you who you are.* IEEE Transactions on Information Forensics and Security, 2014. **9**(3): p. 364-374.
2. Özkan, K., K. Zühal, and S. Erol, *An image-based recommender system based on image annotation.* European Journal of Engineering and Natural Sciences, 2019. **3**(1): p. 12-16.
3. Lei, C., et al. *Comparative deep learning of hybrid representations for image recommendations*. in *Proceedings of the IEEE conference on computer vision and pattern recognition*. 2016.
4. Zhang, J., et al., *Personalized recommendation of social images by constructing a user interest tree with deep features and tag trees.* IEEE Transactions on Multimedia, 2019. **21**(11): p. 2762-2775.

5. Batmaz, Z., et al., *A review on deep learning for recommender systems: challenges and remedies.* Artificial Intelligence Review, 2019. **52**: p. 1-37.
6. Geng, X., et al. *Learning image and user features for recommendation in social networks*. in *Proceedings of the IEEE international conference on computer vision*. 2015.
7. Guntuku, S.C., et al., *Understanding deep representations learned in modeling users likes.* IEEE Transactions on Image Processing, 2016. **25**(8): p. 3762-3774.
8. Shani, G., et al., *An MDP-based recommender system.* Journal of Machine Learning Research, 2005. **6**(9).
9. Taghipour, N. and A. Kardan. *A hybrid web recommender system based on q-learning*. in *Proceedings of the 2008 ACM symposium on Applied computing*. 2008.
10. Zheng, G., et al. *DRN: A deep reinforcement learning framework for news recommendation*. in *Proceedings of the 2018 world wide web conference*. 2018.
11. Zhao, X., et al. *Recommendations with negative feedback via pairwise deep reinforcement learning*. in *Proceedings of the 24th ACM SIGKDD international conference on knowledge discovery & data mining*. 2018.
12. Xiangyu, Z., et al., *Deep reinforcement learning for list-wise recommendations.* arXiv preprint arXiv:1801.00209, 2017.
13. Hu, Y., et al. *Reinforcement learning to rank in e-commerce search engine: Formalization, analysis, and application*. in *Proceedings of the 24th ACM SIGKDD international conference on knowledge discovery & data mining*. 2018.
14. Dulac-Arnold, G., et al., *Reinforcement learning in large discrete action spaces. corr abs/1512.07679 (2015).* arXiv preprint arXiv:1512.07679, 2015.
15. Afsar, M.M., T. Crump, and B. Far, *Reinforcement learning based recommender systems: A survey.* ACM Computing Surveys, 2022. **55**(7): p. 1-38.
16. Liu, F., et al., *Deep reinforcement learning based recommendation with explicit user-item interactions modeling.* arXiv preprint arXiv:1810.12027, 2018.
17. Zhang, W., et al., *Recommendation system in social networks with topical attention and probabilistic matrix factorization.* PloS one, 2019. **14**(10): p. e0223967.
18. Bobadilla, J., et al., *Recommender systems survey.* Knowledge-based systems, 2013. **46**: p. 109-132.
19. Ahmadkhani, S. and M.E. Moghaddam, *An image recommendation technique based on fuzzy inference system.* Soft Computing, 2021. **25**(8): p. 6427-6443.
20. Ahmadkhani, S. and M.E. Moghaddam, *Image recommender system based on compact convolutional transformer image style recognition.* Journal of Electronic Imaging, 2022. **31**(4): p. 043054-043054.
21. Zhang, H., et al., *Learning from collective intelligence: Feature learning using social images and tags.* ACM transactions on multimedia computing, communications, and applications (TOMM), 2016. **13**(1): p. 1-23.
22. Yu, J. and L. Zhao, *A novel deep CNN method based on aesthetic rule for user preferential images recommendation.* Journal of Applied Science and Engineering, 2021. **24**(1): p. 49-55.
23. Zhang, J., et al., *Personalized social image recommendation method based on user-image-tag model.* IEEE Transactions on Multimedia, 2017. **19**(11): p. 2439-2449.
24. Huang, L., et al., *A deep reinforcement learning based long-term recommender system.* Knowledge-Based Systems, 2021. **213**: p. 106706.
25. Zhao, X., et al. *Deep reinforcement learning for page-wise recommendations*. in *Proceedings of the 12th ACM conference on recommender systems*. 2018.
26. Zhao, S., et al. *Exploring principles-of-art features for image emotion recognition*. in *Proceedings of the 22nd ACM international conference on Multimedia*. 2014.
27. Machajdik, J. and A. Hanbury. *Affective image classification using features inspired by psychology and art theory*. in *Proceedings of the 18th ACM international conference on Multimedia*. 2010.
28. You, Q., et al. *Building a large scale dataset for image emotion recognition: The fine print and the benchmark*. in *Proceedings of the AAAI conference on artificial intelligence*. 2016.
29. Sun, M., et al. *Discovering affective regions in deep convolutional neural networks for visual sentiment prediction*. in *2016 IEEE International Conference on Multimedia and Expo (ICME)*. 2016. IEEE.
30. Tan, M. and Q. Le. *Efficientnet: Rethinking model scaling for convolutional neural networks*. in *International conference on machine learning*. 2019. PMLR.
31. Guntuku, S.C., S. Roy, and L. Weisi. *Personality modeling based image recommendation*. in *MultiMedia Modeling: 21st International Conference, MMM 2015, Sydney, NSW, Australia, January 5-7, 2015, Proceedings, Part II 21*. 2015. Springer.
32. Guntuku, S.C., et al., *Who likes what, and why? insights into personality modeling based on imagelikes'.* IEEE Transactions on Affective Computing, 2016. **3045**(c): p. 1.
33. Segalin, C., D.S. Cheng, and M. Cristani, *Social profiling through image understanding: Personality inference using convolutional neural networks.* Computer Vision and Image Understanding, 2017. **156**: p. 34-50.
34. Matsuo, S. and K. Yanai. *CNN-based style vector for style image retrieval*. in *Proceedings of the 2016 ACM on International Conference on Multimedia Retrieval*. 2016.
35. Chang, W.-Y., Y.-R. Yeh, and Y.-C.F. Wang. *Style-oriented landmark retrieval and summarization*. in *2016 Asia-Pacific Signal and Information Processing Association Annual Summit and Conference (APSIPA)*. 2016. IEEE.

36. Chu, W.-T. and Y.-L. Wu, *Image style classification based on learnt deep correlation features.* IEEE Transactions on Multimedia, 2018. **20**(9): p. 2491-2502.
37. Vaswani, A., et al., *Attention is all you need.* Advances in neural information processing systems, 2017. **30**.
38. Karayev, S., et al., *Recognizing image style.* arXiv preprint arXiv:1311.3715, 2013.
39. Russakovsky, O., et al., *Imagenet large scale visual recognition challenge.* International journal of computer vision, 2015. **115**: p. 211-252.

Figure

Click here to access/download;Figure;Fig1.tif

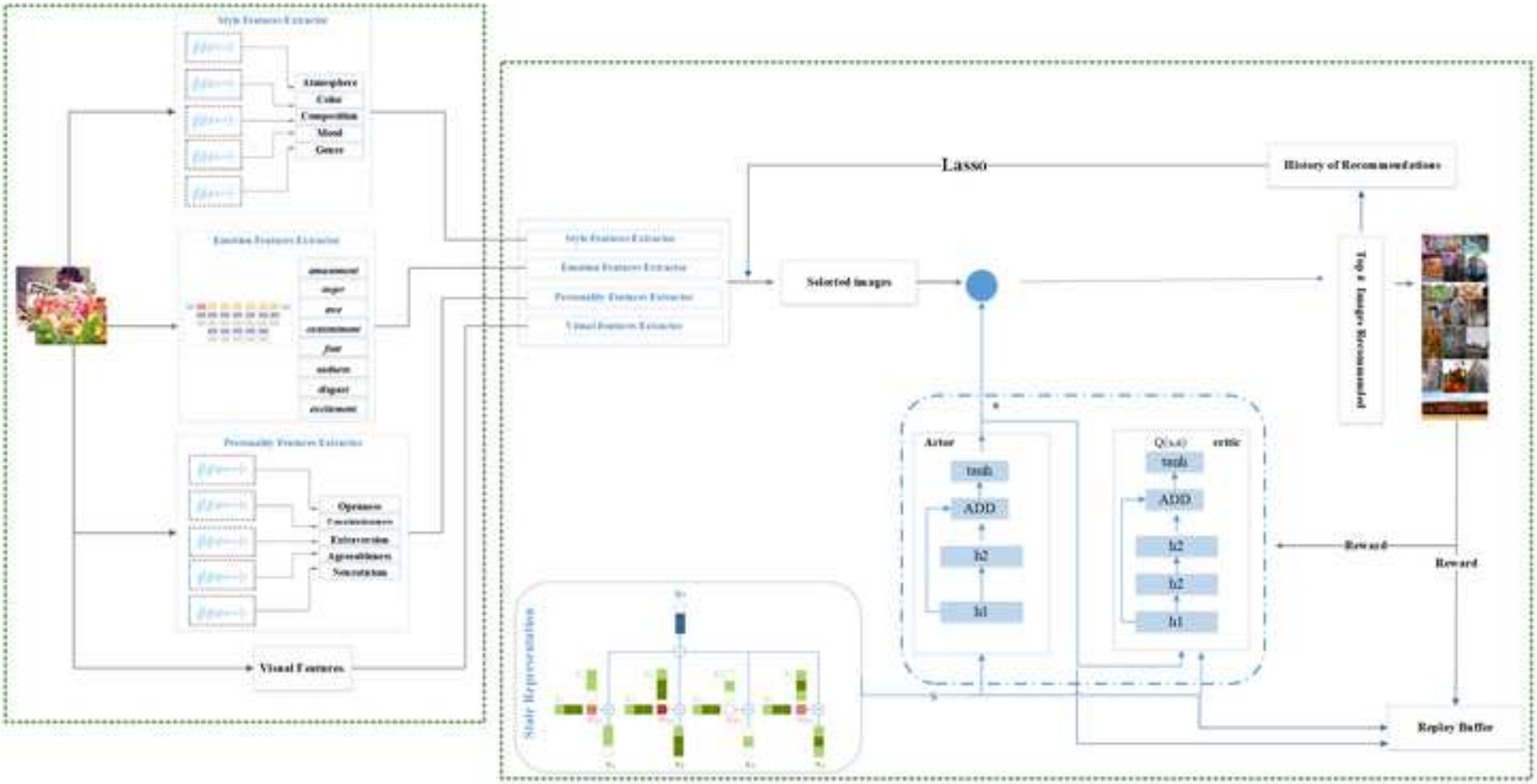

Figure

Click here to access/download;Figure;Fig2.tif

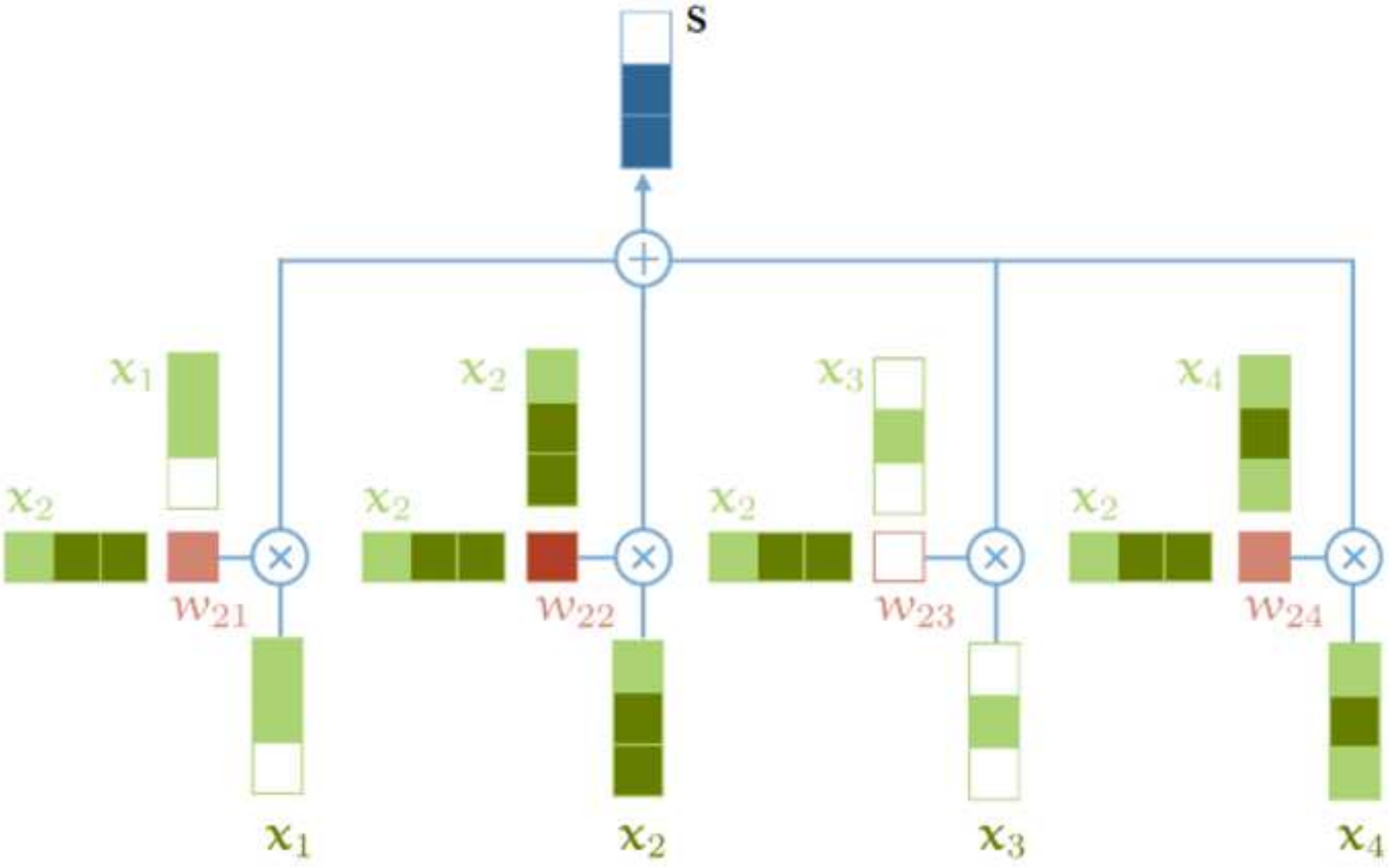

Figure Click here to access/download;Figure;Fig3.tif

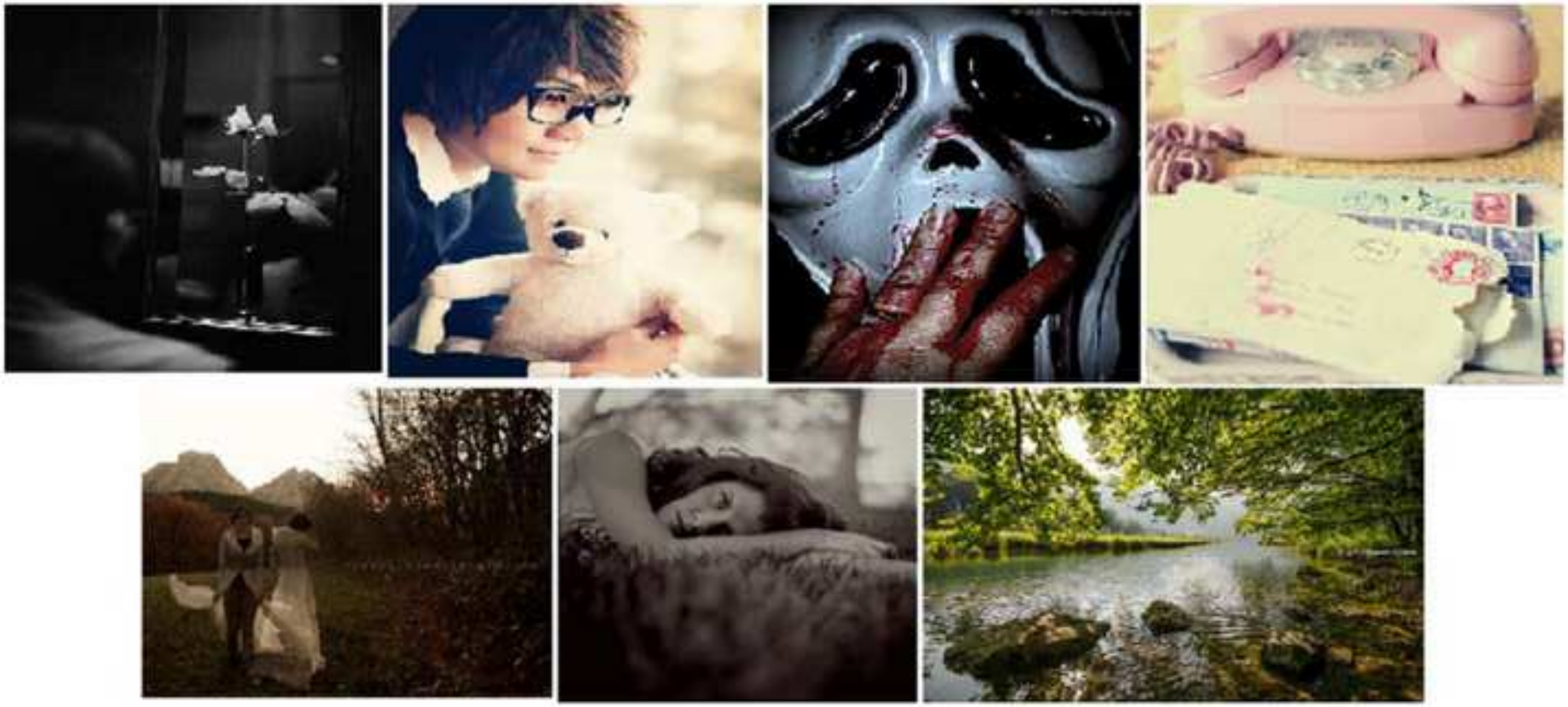

Figure Click here to access/download;Figure;Fig4.tif

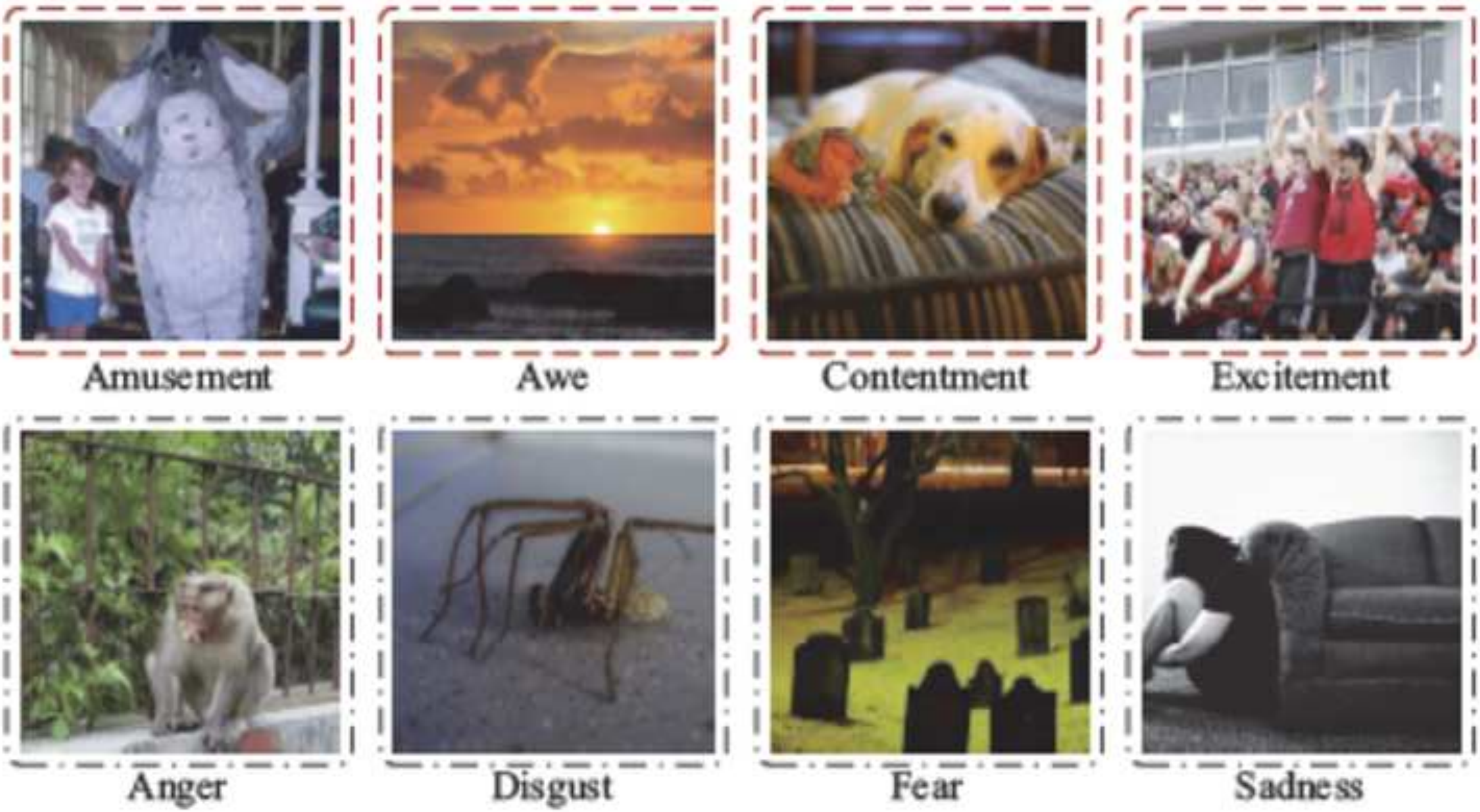

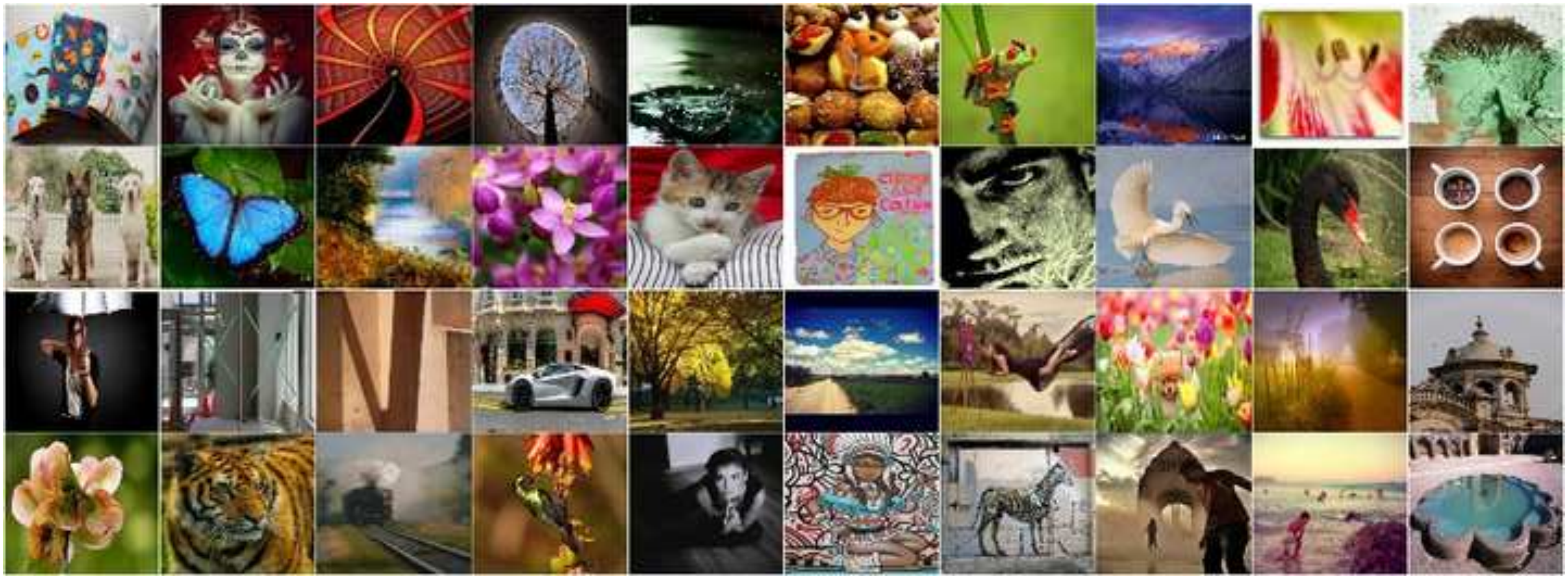

 Click here to access/download;Figure;Fig6.tif

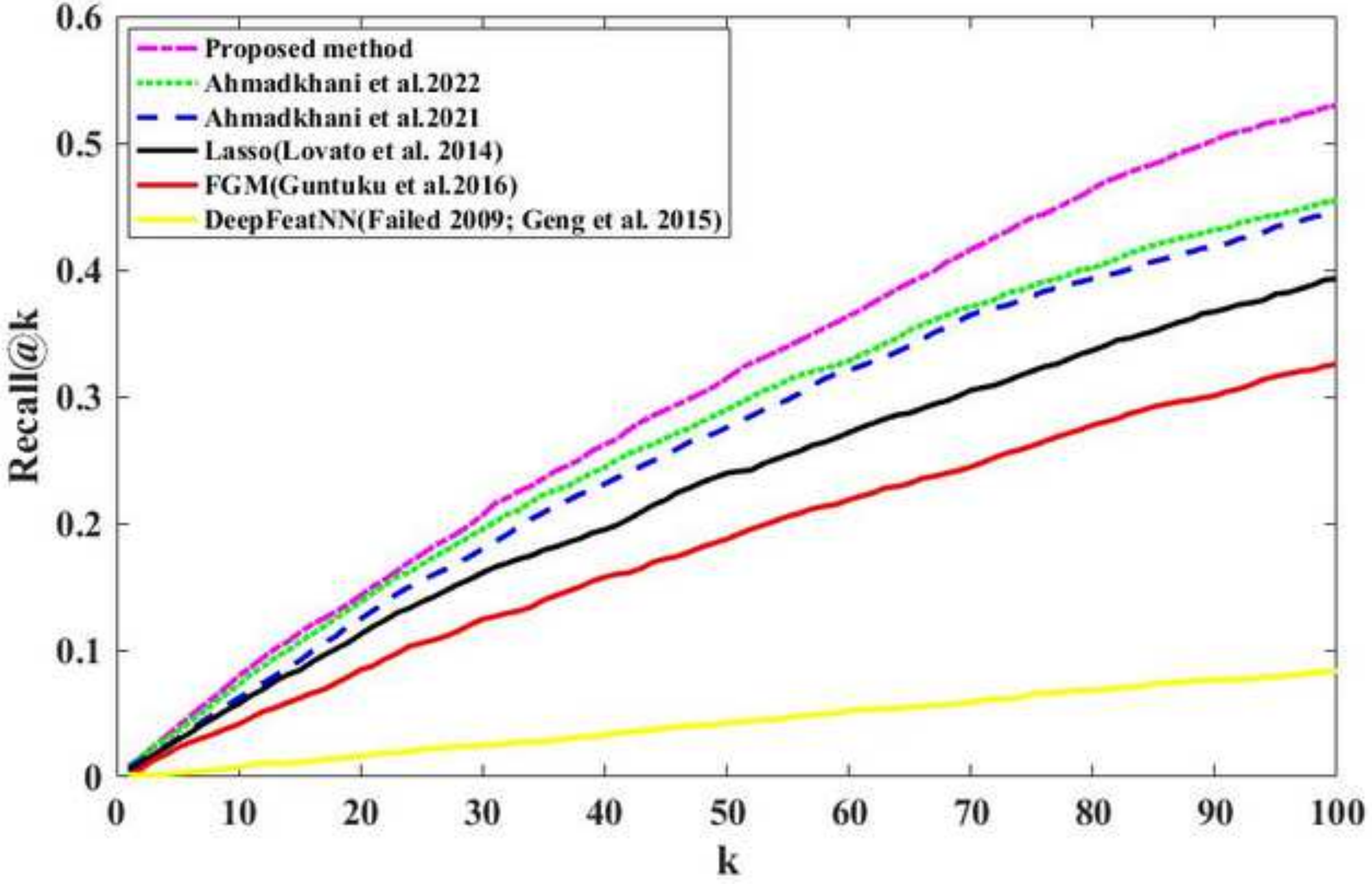

Figure Click here to access/download;Figure;Fig7.tif

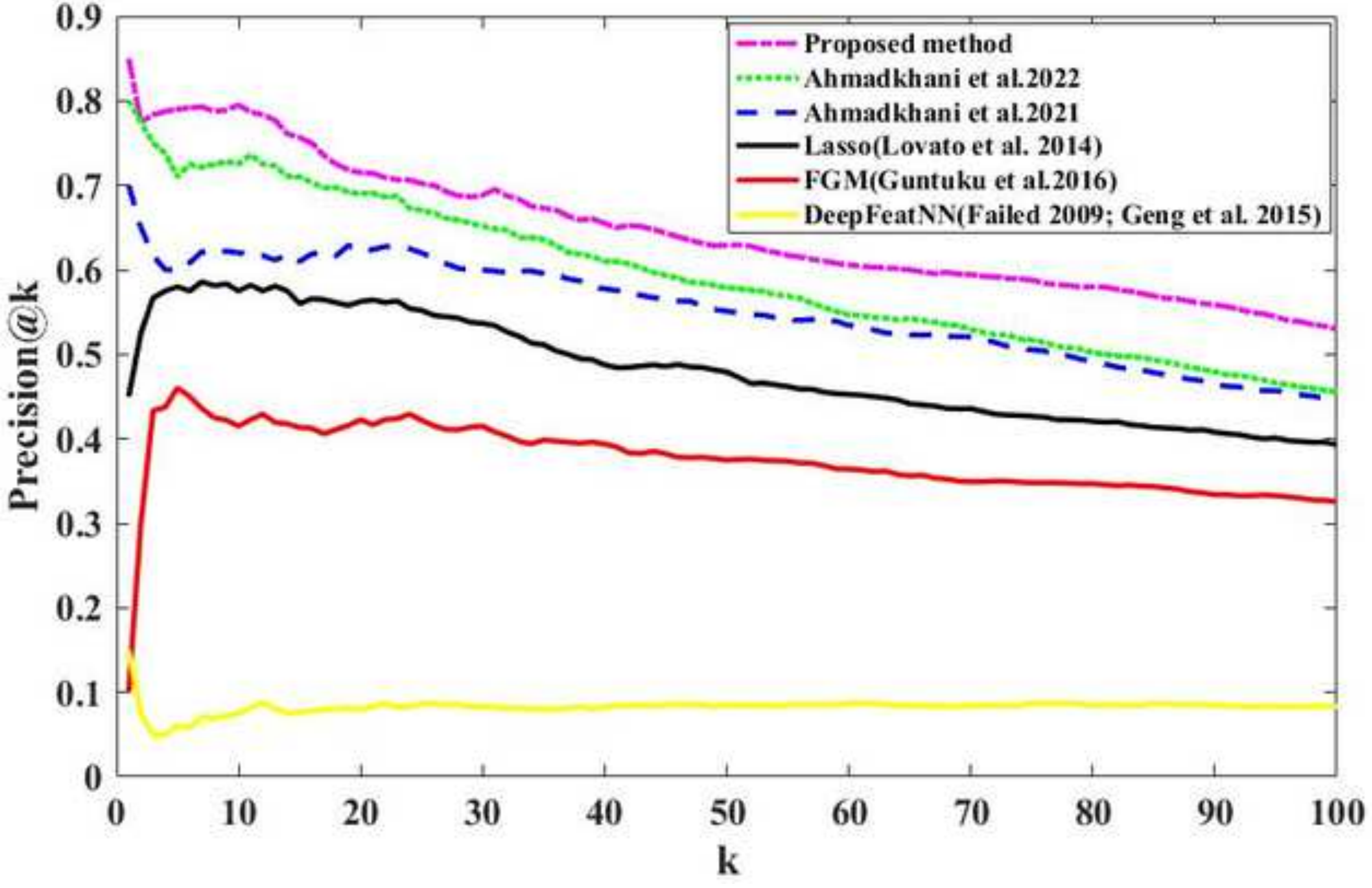

Figure
Click here to access/download;Figure;Fig8.tif

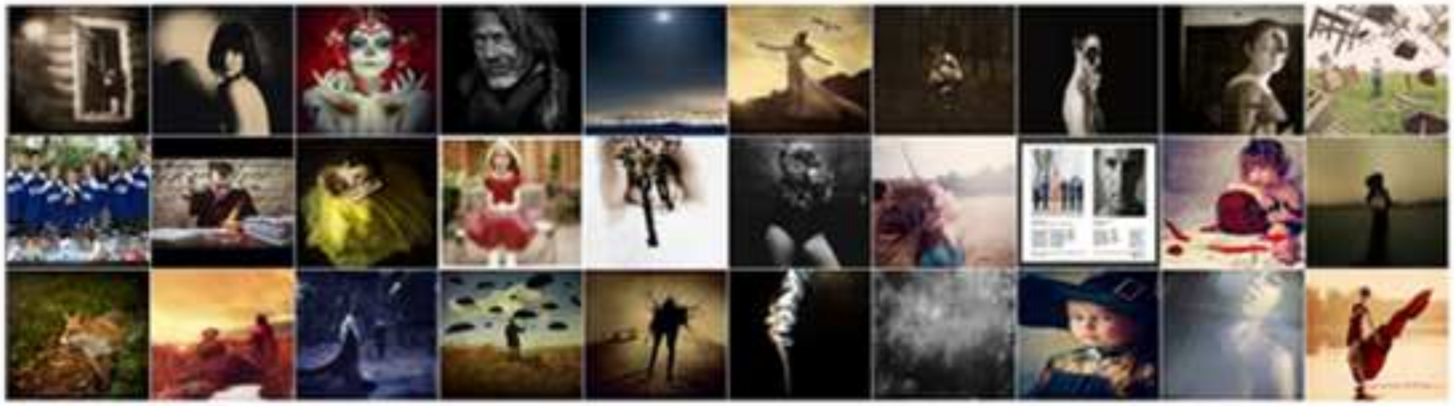

(a) Sample of the User2 Training images

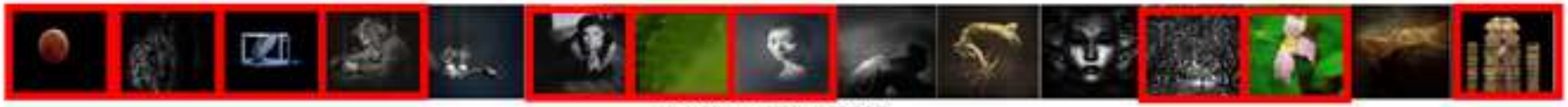

(b) VisualFeat.FGM

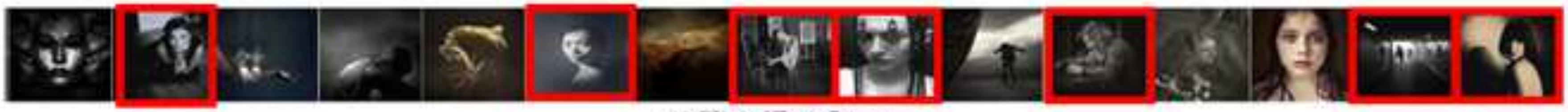

(c) VisualFeat.Lasso

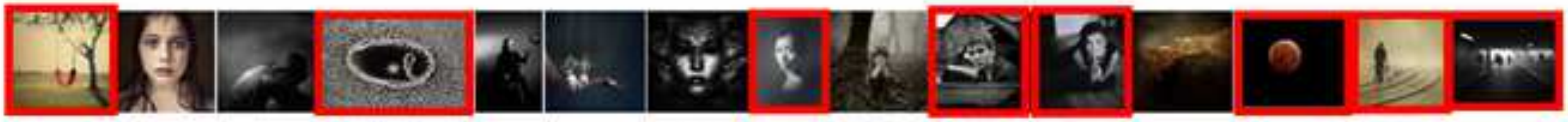

d) VOFeatFGMPFIS

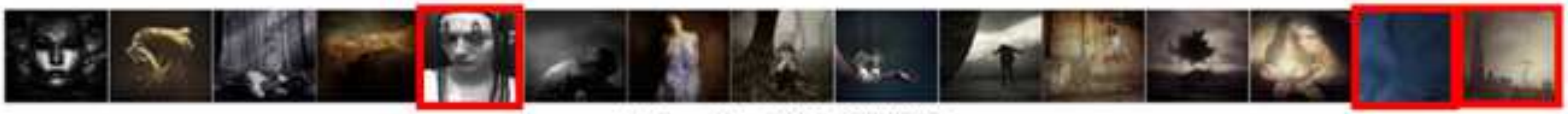

(e) VisualFeat.FTFMCT.FGM

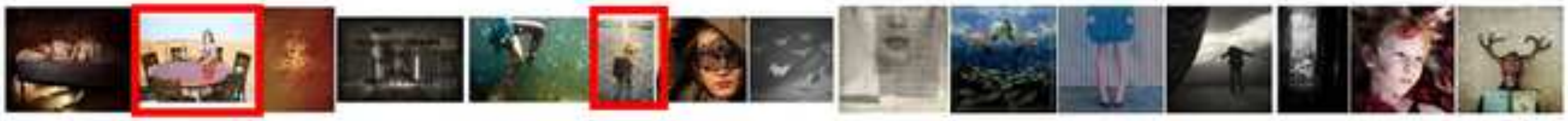

f) Porposed method
