# Supplementary material for: A social image recommendation system based on deep reinforcement learning
Source: PLoS One. 2024 Apr 4;19(4):e0300059. doi: 10.1371/journal.pone.0300059 (PMC10994284; doi:10.1371/journal.pone.0300059)
Supplement: Attachment — Submitted filename: review.docx [file pone.0300059.s002.docx]

This paper introduced a social image recommendation system through reinforcement learning method. The authors introduce the current development of recommender system and its corresponding related works. Thereafter, they vividly depict the proposed method including numerous components. Then, they conducted some experiments on four datasets comparing with some baselines. Finally, they draw a conclusion that the proposed method makes progress among some previous image recommendation methods.

**Innovative points of the proposed method are as follows.**

1. They apply pretrained image feature extractors in reinforcement learning based image recommendation tasks.
2. They proposed a new state representation method for image recommendation.
3. Last but not least, the vector representing user interest is applied to better capture user preference.

**Some weaknesses also emerge which are as follows.**

1. Some formula and equations are presented in the form of pictures (formula (5), formula (6)), which ought to be replaced by latex generated formula.
2. Some images claiming the core structure of the proposed method is presented in the form of bitmap (fig 1. and fig 2.). I don’t know if it is the transfer problem between docx file and pdf file. But in my opinion, such pictures are recommended to be presented in the form of vector graphics. Moreover, the layout of fig 1. is too sparse. Some components of the image lie too far between each other, it’s recommended to be re-arranged.
3. Experiments seems Inadequate. Some parameter/hyper-parameter analysis or ablation study may be conducted to prove the robustness and effectiveness of different components in the whole algorithm procedure.
4. The methods introduced in chapter 3-1) are some manually feature extractors. In the era of deep learning, many DL methods which can automatically capture implicit features, from my point of view, may be tried to get better performance. Manual feature setting should be avoided as much as possible. Such progress may be introduced in future works.
5. The methods introduced in chapter 3-2-1) named ‘self-attention’ is not the ‘self-attention’ in the present sense. It has no trainable parameter. I think a trainable self-attention module is more reasonable. Such progress may be introduced in future works.

**To be concluded,** the proposed work is considered to be slightly innovated by combining different methods together. But the outcome is proved to be impressive. Some faults exist in the paper which should be removed. **The paper needs minor revision.**
