# Supplementary material for: A social image recommendation system based on deep reinforcement learning
Source: PLoS One. 2024 Apr 4;19(4):e0300059. doi: 10.1371/journal.pone.0300059 (PMC10994284; doi:10.1371/journal.pone.0300059)
Supplement: Attachment — Submitted filename: Response to Reviewers-2-1.docx [file pone.0300059.s004.docx]

**Dear respectful editor and reviewers,**

**We would like to appreciate your comments to improve the paper quality. We tried our best to address all the comments. Hope that the revised paper satisfies the publications’ requirements.**

**Sincerely,**

**Corresponding author**

**Response to Reviewers Comment**

Reviewer #1

**Reviewer #1:** This paper presents a dynamic image recommender system utilizing deep reinforcement learning and incorporating novel features like emotion, style, and personality. Authors introduce a new method for state representation in reinforcement learning to overcome this limitation. Experimental results demonstrate that proposed approach significantly enhances Recall and Precision for personalized image recommendations. Below are some my concern:

**Comment-** **The language used throughout the manuscript needs to be improved.**

**Response: The language of the manuscript was reviewed and grammatical errors were corrected.**

**Comment-** **Improve the introduction part by making it more concise.**

**Response: To respond to this comment, the Introduction section has been rewritten in the revised manuscript as follows:**

**Line 18:**

*“In recent years, with development of the Internet and social networks, impressive research effort has been directed at recommender systems. This is because, in the face of the huge volume and variety of information, we need a system that can automatically identify and cater to the user's interests. Despite the various advances in recommender systems, existing ones still require further improvements to provide more efficient recommendations that are applicable to a broader range of applications. The exponential increase in images as an important source of information in online services and social networks prompted us to investigate the image-based recommender systems of social networks.*

*Prior studies have explored handcrafted features [1, 2], as well as deep learning models [3-5] for image representation and recommendation. Various similarity measures [6] and classification methods [7] have also been employed. The remarkable point in all of this is that the recommendation is often viewed as a static procedure, assuming that the user’s underlying preferences remain unchanged. In reality, personalizing image recommendations according to individual user preferences is a complex task. User preferences evolve over time, necessitating a dynamic interaction between the system and the user, thereby indicating the need for a dynamic recommendation process. Also, if there is limited data available, it is difficult to make accurate recommendations, which is referred to as the cold start problem.*

*Reinforcement learning is increasingly used for its ability to tackle complex problems involving dynamic modeling and long-term planning [8]. The use of reinforcement learning in recommender systems is not new and has been used in old works. Model-based RL techniques such as POMDP [9] and Q-learning [10] were among the first RL techniques for modeling recommendation methods. However, when the number of proposed items is large, these methods cannot be efficient due to the time complexity. Later, model-free RL techniques were also used for recommendation. They can be categorized into two groups: value-based [11-13] and policy-based [13-15].*

*Although using RL for recommender systems is not new, mainly because of scalability problems, traditional RL algorithms were not very practical. With the advent of deep reinforcement learning (DRL), a new trend emerged in this field that enabled the application of RL to solve recommendation problems with large state and action spaces [16].*

*For example, the authors in [17] propose a recommendation framework based on deep reinforcement learning. They model the interactions between the users and recommender systems using an Actor-Critic reinforcement learning scheme and consider both dynamic adaptation and long-term rewards. Xiangyu et al. [13] used deep reinforcement learning to automatically learn optimal recommendation strategies and model the recommendation as a Markov decision process.*

*Due to the operationally of recommender systems based on reinforcement learning for real-world problems, we propose using deep Reinforcement Learning to define the sequential interactions between users and the recommender system for the automatic learning of optimal strategies from user feedback.*

*The proposed method consists of two main parts, and there are innovations in each part. In the first part, we propose to extract a set of different features from images that represent the characteristics and preferences of the user. To this end, we use three new components: emotion analysis, personality recognition, and style detection to represent users and improve the social image recommender system. In the second part, we propose a new framework based on actor-critic reinforcement learning and with a state representation module to have a dynamic recommender system.*

*For this study the main contributions can be summarized as follows:*

*1) Propose an image recommender system in a new deep reinforcement learning framework*

*2) Introducing a new method for state representation.*

*3) Introducing three components (style, Emotion, personality) that can be useful in images recommendation to create user's characteristics vector and investigate its effect on the recommended images on each user.*

*The structure of this paper is as follows: related works and background are presented in Section 2. The proposed methods are introduced in Section 3. Experimental details and results are discussed in Section 4. Finally, we conclude this paper in Section 5 and discuss some future work.”*

**Comment-** **More discussion on the current limitations on machine learning techniques can be helpful.**

**Response: We discuss the current limitations as follows:**

**Line 24:**

*“Prior studies have explored handcrafted features [1, 2], as well as deep learning models [3-5] for image representation and recommendation. Various similarity measures [6] and classification methods [7] have also been employed. The remarkable point in all of this is that the recommendation is often viewed as a static procedure, assuming that the user’s underlying preferences remain unchanged. In reality, personalizing image recommendations according to individual user preferences is a complex task. User preferences evolve over time, necessitating a dynamic interaction between the system and the user, thereby indicating the need for a dynamic recommendation process. Also, if there is limited data available, it is difficult to make accurate recommendations, which is referred to as the cold start problem.”*

**Comment-** **Detailed analysis and more results should be added.**

**Response: In response to the comment, we have augmented the revised manuscript with further analysis and findings. The performance of the proposed framework is now evaluated using the F1@k and NDCG@k metrics, along with the previously included Recall@k and Precision@k. The results of these evaluations are illustrated in Figures 6 to 9.**

**Additionally, we have conducted a comprehensive ablation study to ascertain the robustness and efficacy of the various components within our algorithm. The specifics of this study are depicted in Figures 11 and 12.**

**Consequently, the revised manuscript has been updated as described above.**

**Line 420:**

*“After training, for each user, the test images are ranked based on the score given by the recommendation model. We implement different methods and report the top k recommended images, where k is adjustable. We know only the relevant images (user “likes”) for each users. We used the following metrics to evaluate recommendation results.*

***Recall@k:*** *this means the proportion of relevant images in the top k. The formulation of Recall@k is as (Eq. 10)*

***Precision@k:*** *means the proportion of relevant top k images, the formulation of Precision@k is as (Eq. 11).*

***NDCG@k:*** *The Normalized Discounted Cumulative Gain (NDCG) measures the relevance of the recommended images and is defined as (Eq 12). Where IDCG@k is the maximum NDCG@k that corresponds to the optimal ranking list so that the perfect NDCGk is 1.*

***F1@K:*** *The F1 Score is a composite metric that incorporates both Precision and Recall using the harmonic mean. In the context of recommendation systems, this F1 Score operates the same way as the regular version ((Eq. 13)).*

$Recall=\frac{Relevant\_Items\_Recommended in topk}{Relevant\_Items}$ *(10)*

$Precision=\frac{Relevant\_Items\_Recommended in topk}{k\_Items\_Recommended}$ *(11)*

$NDCG@k= \frac{1}{IDCG@k}\times\sum_{i=1}^{k} \frac{G}{{log}_{2} (i+1)}$	*(12)*

$F1@K= \frac{2\times Precision@K \times Recall@K}{Precision@K+Recall@K}$	*(13)*

**Fig. 6 Recall@k for different k values**

**Fig. 7 Precision@k for different k values**

**Fig. 8 NDCG@k for different k values**

**Fig. 9 F1@k for different k values**

”

**Line 499:**

*“In this paper we proposed a dynamic Image recommender system using deep reinforcement learning frame work by employing a set of new extracted features includes emotion, style, and personality features. We claimed that using these components in the proposed framework improves the results. Therefore, the ablation study was designed to systematically evaluate the contribution of each component of our proposed framework. By considering one component at a time, we were able to assess its impact on the overall performance. This study has provided valuable insights into how each element contributes to the efficacy of our method. To this end, we considered three approaches:*

1. *In the first approach, we use visual and style features within the proposed framework to investigate the effect of style features on recommendations (This is shown as (****VS****) in the Fig 12 and 13).*
2. *In the second approach, we apply visual and emotion features to examine the impact of emotion features (This is shown as (****VE****) in the Fig 12 and 13).*
3. *In the third approach, we use visual and personality features within the proposed framework to study the influence of personality features on recommendations (This is shown as (****VP****) in the Fig 12 and 13).*

*We calculate Recall@K and Precision@K for all users using Eq. (10) and (11) for each approach. The results are shown in Fig. 12. The top row displays Recall@K for the three approaches, while the bottom row presents Precision@K. The results underscore the importance of these components within our method. These results indicate that incorporating each of these three components into the proposed framework improves performance. The ablation study outcomes strongly support our initial hypotheses and assertions about our proposed framework. Additionally, Figure 13 compares the effectiveness of each of these three approaches with the proposed method, which utilizes a combination of all three components within a deep reinforcement learning framework.*

***Fig. 11: shows the results for three approaches: The top row displays Recall@K, and the bottom row shows Precision@K for different k values.***

***Fig. 12 the comparison between the three approaches and the proposed method***

***”***

**Comment-** **Include in conclusion session more future suggestions.**

**Response: To respond to this comment, the CONCLUSION section has been rewritten in the revised manuscript as follow:**

**Line 568:**

*“For future work exploring additional behavioral features related to user behavior is possible. Methods that enhance accuracy in detecting emotions, style, and personality can be investigated, potentially improving the recommender system. We have utilized a combination of manual features and those obtained using deep learning models. Future research could explore other deep learning methods capable of automatically capturing implicit features to achieve better performance. Additionally, self-attention, the core mechanism of the Transformer, focuses on measuring dependencies within a sequence's components to gain a more accurate understanding of the entire sequence. In our work, we employed this concept to calculate the new state. Our approach does not have any trainable parameters, which could be considered in future research."*

**Comment-** **Include the more comparison metrics.**

**Response: To evaluate the performance of the proposed framework, in addition to Recall@k and Precision@k, we select F1@K and NDCG@k as the metrics to measure the performance.**

**Line 420:**

*“After training, for each user, the test images are ranked based on the score given by the recommendation model. We implement different methods and report the top k recommended images, where k is adjustable. We know only the relevant images (user “likes”) for each users. We used the following metrics to evaluate recommendation results.*

***Recall@k:*** *this means the proportion of relevant images in the top k. The formulation of Recall@k is as (Eq. 10)*

***Precision@k:*** *means the proportion of relevant top k images, the formulation of Precision@k is as (Eq. 11).*

***NDCG@k:*** *The Normalized Discounted Cumulative Gain (NDCG) measures the relevance of the recommended images and is defined as (Eq 12). Where IDCG@k is the maximum NDCG@k that corresponds to the optimal ranking list so that the perfect NDCGk is 1.*

***F1@K:*** *The F1 Score is a composite metric that incorporates both Precision and Recall using the harmonic mean. In the context of recommendation systems, this F1 Score operates the same way as the regular version ((Eq. 13)).*

$Recall=\frac{Relevant\_Items\_Recommended in topk}{Relevant\_Items}$ (10)

$Precision=\frac{Relevant\_Items\_Recommended in topk}{k\_Items\_Recommended}$ (11)

$NDCG@k= \frac{1}{IDCG@k}\times\sum_{i=1}^{k} \frac{G}{\log_{2} (i+1)}$	(12)

$F1@K= \frac{2\times Precision@K \times Recall@K}{Precision@K+Recall@K}$	(13)

**Fig. 6 Recall@k for different k values**

**Fig. 7 Precision@k for different k values**

**Fig. 8 NDCG@k for different k values**

**Fig. 9 F1@k for different k values**

”

**Reviewer #2:** **This paper introduced a social image recommendation system through reinforcement learning method. The authors introduce the current development of recommender system and its corresponding related works. Thereafter, they vividly depict the proposed method including numerous components. Then, they conducted some experiments on four datasets comparing with some baselines. Finally, they draw a conclusion that the proposed method makes progress among some previous image recommendation methods.**

**Innovative points of the proposed method are as follows.**

**1. They apply pretrained image feature extractors in reinforcement learning based image recommendation tasks.
2. They proposed a new state representation method for image recommendation.**

**3. Last but not least, the vector representing user interest is applied to better capture user preference.**

**Some weaknesses also emerge which are as follows.**

**Comment-** **Some formula and equations are presented in the form of pictures (formula (5), formula (6)), which ought to be replaced by latex generated formula.**

**Response:**

**We have addressed the concern regarding the presentation of formulas (5) and (6). They have now been rewritten using Microsoft Word's Equation Tool for clarity, consistency, and improved integration with the rest of the document. This modification ensures that the formulas are now properly formatted, and accessible.**

**Line 297:**

$s= \sum_{j} w_{ij}x_{j}$	(7)

**Line 300:**

$w_{ij}^{'}=x_{i}^{T}x_{j}$	(8)

**Line 304:**

$w_{ij}=\frac{exp w_{ij}^{'}}{\sum_{j} \exp w_{ij}^{'}}$	(9)

**Comment- Some images claiming the core structure of the proposed method is presented in the form of bitmap (fig 1. and fig 2.). I don’t know if it is the transfer problem between docx file and pdf file. But in my opinion, such pictures are recommended to be presented in the form of vector graphics. Moreover, the layout of fig 1. is too sparse. Some components of the image lie too far between each other, it’s recommended to be re-arranged.**

**Response:**

**Thank you for pointing out the issues with Figures 1 and 2 in our manuscript. We have taken the following steps to address this issues:**

**To address this, we have converted both figures from bitmap images to vector graphics. This change ensures higher quality and clarity, especially when scaling for different sizes. The figures are now sharper, more professional in appearance, and better suited for high-quality printing and viewing in various digital formats.**

**Improvement in Layout of Figure 1: In response to your feedback on the layout of Figure 1 being too sparse, we have re-arranged the components of the image. The revised layout is more compact and aesthetically pleasing, with a better visual balance of the components.**

**Line 305:**

**Fig. 2 State representation using self-attention**

**Line 117:**

**Fig. 1 Workflow of the proposed method (The images shown as input and output in this workflow are from reference [1]).**

**Comment- Experiments seems Inadequate. Some parameter/hyper-parameter analysis or ablation study may be conducted to prove the robustness and effectiveness of different components in the whole algorithm procedure.**

**Response: In light of your suggestions, we have now conducted a comprehensive ablation study to investigate the robustness and effectiveness of the various components of our algorithm. The details of this study are outlined below:**

**Line 499:**

*“In this paper we proposed a dynamic Image recommender system using deep reinforcement learning frame work by employing a set of new extracted features includes emotion, style, and personality features. We claimed that using these components in the proposed framework improves the results. Therefore, the ablation study was designed to systematically evaluate the contribution of each component of our proposed framework. By considering one component at a time, we were able to assess its impact on the overall performance. This study has provided valuable insights into how each element contributes to the efficacy of our method. To this end, we considered three approaches:*

1. *In the first approach, we use visual and style features within the proposed framework to investigate the effect of style features on recommendations (This is shown as (****VS****) in the Fig 12 and 13).*
2. *In the second approach, we apply visual and emotion features to examine the impact of emotion features (This is shown as (****VE****) in the Fig 12 and 13).*
3. *In the third approach, we use visual and personality features within the proposed framework to study the influence of personality features on recommendations (This is shown as (****VP****) in the Fig 12 and 13).*

*We calculate Recall@K and Precision@K for all users using Eq. (10) and (11) for each approach. The results are shown in Fig. 12. The top row displays Recall@K for the three approaches, while the bottom row presents Precision@K. The results underscore the importance of these components within our method. These results indicate that incorporating each of these three components into the proposed framework improves performance. The ablation study outcomes strongly support our initial hypotheses and assertions about our proposed framework. Additionally, Figure 13 compares the effectiveness of each of these three approaches with the proposed method, which utilizes a combination of all three components within a deep reinforcement learning framework.*

***Fig. 11: shows the results for three approaches: The top row displays Recall@K, and the bottom row shows Precision@K for different k values.***

***Fig. 12 the comparison between the three approaches and the proposed method***

***”***

**In addition, there are parameters in the proposed method, must be adjusted which is described in the revised manuscript as follow:**

**Line 447:**

*“In our experiments, we conduct 400 episodes using the proposed method. In each episode, 100 unique images are recommended to the user; we ensure that items already recommended in previous episodes are not repeated by removing them from the candidate set. Additionally, to make decisions based on the users' most recent interactions, we store up to 100 images (regardless of user liked or not) recommended in each episode, forming what we refer to as the 'recommendation history'. The discount rate (γ) is set at 0.3. For all reinforcement learning-based methods, we employ the Adam optimizer. The mini-batch size is configured at 10, and the initial learning rate is set to 0.001. We determine the optimal parameters, including the hidden layer sizes for both the actor and critic networks, through grid search.”*

**Comment- The methods introduced in chapter 3-1) are some manually feature extractors. In the era of deep learning, many DL methods which can automatically capture implicit features, from my point of view, may be tried to get better performance. Manual feature setting should be avoided as much as possible. Such progress may be introduced in future works.**

**Response: We have used some of the manual features along with features obtained using deep learning models. For style detection, personality analysis, and emotion detection we use three different deep-learning methods. In future works, the use of other deep learning methods that can automatically capture implicit features, to get better performance, can be investigated.**

**Line 565:**

*“Furthermore, we employ a set of newly extracted features, such as emotion, style, and personality, to enhance efficiency. In Section 4, the results of our tests demonstrate how the use of these components improves outcomes. Overall, the results indicate that our proposed method significantly outperforms some related works in personalized image recommendation. For future work exploring additional behavioral features related to user behavior is possible. Methods that enhance accuracy in detecting emotions, style, and personality can be investigated, potentially improving the recommender system. We have utilized a combination of manual features and those obtained using deep learning models. Future research could explore other deep learning methods capable of automatically capturing implicit features to achieve better performance.”*

**Comment- The methods introduced in chapter 3-2-1) named ‘self-attention’ is not the ‘self-attention’ in the present sense. It has no trainable parameter. I think a trainable self-attention module is more reasonable. Such progress may be introduced in future works.**

**Response: Self-attention is the central mechanism of the Transformer. The purpose of Self-Attention is to measure the dependencies of the components in a sequence with each other in order to have a more accurate perception of the whole sequence. In our work, we used this idea and calculate the new state. Our approach has no trainable parameter and it can be consider in future works.**

**Line 573:**

*“Additionally, self-attention, the core mechanism of the Transformer, focuses on measuring dependencies within a sequence's components to gain a more accurate understanding of the entire sequence. In our work, we employed this concept to calculate the new state. Our approach does not have any trainable parameters, which could be considered in future research.”*

**To be concluded, the proposed work is considered to be slightly innovated by combining different methods together. But the outcome is proved to be impressive. Some faults exist in the paper which should be removed. The paper needs minor revision.**

**Reviewer #3:**

**Comment-** **The paper need proofreading and editing to reach Standard English.**

**Response: The language of the manuscript was reviewed and grammatical errors were corrected.**

We employed advanced grammar and spell-check tools also to ensure the correctness and clarity of the text. These comprehensive revisions have greatly improved the readability and professionalism of our manuscript.

**Comment-** **Please refer to the attached file to get my comments!**

**Thank you for taking the time to review the manuscript file and provide your valuable feedback. We appreciate your input, and we have carefully considered all of your comments. Below, we have provided explanations and responses to each of the points you raised:**

**Line5: what is the meaning of dynamic information here?**

**Response:**

**Dynamic information refers to data, content, or information that changes or are updated in real-time or near-real-time continuously. It contrasts with static information, which remains unchanged or is updated infrequently. Dynamic information is prevalent in various domains and is made possible by advances in technology, particularly the internet and digital communication systems.**

**Line 13: please mention the amount of improvements?**

**Response: The following text has been added to the paper for applying this comment.**

**Line 12:** “*The experimental results show that our proposed method compared to some related works significantly improve Recall@k and Precision@k by ∼7%–10% (for 100 top images recommended) for personalized image recommendation.*”

**Line 122: need English editing.**

**Response: In response to your comments, we have undertaken a thorough review and revision of the manuscript to enhance the grammatical accuracy, readability, and overall clarity.**

**Specifically, line 122 has been modified as follows. In the revised manuscript, the phrase that originally appeared on line 122 has been shifted to line 121 due to the changes in the text.**

**Line 121:** *“In our proposed recommender system framework, four key components contribute in feature extraction as shown in Figure 1. These components are: Visual Feature Extractor, Style Feature Extractor, Emotion Feature Extractor, and Personality Feature Extractor. Each of these features is listed in Table 1. Below, we describe the model designed and used for each component. For every image, we extract an emotion vector to express the image's emotions, a style vector to denote the most prominent styles, and a personality vector to represent key personality features. Our interpretation hinges on the concept that a user tags an image as a favorite when it aligns with their interests in terms of emotion, personality, and style. To extract these three distinct features, we initially train a separate model for each. We then produce the corresponding feature vector by inputting an image into each model. Upon extracting these components, we utilize reinforcement learning to introduce a novel social image recommender system.”*

**Line 148: can one consider this suggestion as a new contribution? I think fine-tuned usage in it. Please explain!**

**Response:**

**We affirm that the integration of emotion as a component in our recommender system indeed constitutes a novel aspect of our research. Emotion analysis plays a crucial role in how users interact with and respond to visual content. However, its application in the context of social image recommendation has been limited. Our system uniquely leverages emotion as a key factor in understanding and predicting user preferences.**

**Although, fine-tuning as a technique might not be new in itself, we fine-tune our model for specifically to enhance the accuracy of emotion feature extraction.**

**To apply this comment line 149 Revised as follows:**

**Line 145:**

“The integration of emotion as a component in our recommender system represents a novel aspect of our research. We employ the fine-tuned EfficientNet network to analyze the emotions conveyed by the images.”

**Line 160: Based on [33] I could not find any new contribution in this part of the paper.**

**Response:**

**We do not claim any original contribution in this part of the paper. Our approach is akin to that implemented in [33], where the study models users' personality traits based on the Five Factor Theory. Consequently, we have adopted five distinct binary classifications for each trait. Utilizing CNN networks trained on ImageNet, we fine-tuned these networks and modified the last layer to suit binary classifications for each trait.**

**To apply this comment line 165 Revised as follows:**

Line 155: “Our approach is similar to the one implemented in [38], where the study models users' personality traits based on the Five Factor Theory. Therefore, five distinct binary classifications are considered for each trait. We have considered CNN networks, trained on the ImageNet, fine-tuning the network, and changed the last layer to adapt binary classifications for each trait.”

**Lin 180: 1- some of the mentioned features need more explanations to be understanble. 2- The list of features has extracted from [1]. Is there any investigation on the bstructive and comprehensiveness of them? What is the meaning of “Color name” feature?**

**Response: to response, we have expanded our descriptions in the manuscript.**

**Line 172: *“****Visual features in Table 1 are part of the features used in [1]. This list of features represents a wide, though not exhaustive, spectrum that is useful for our purpose. It takes into account cues focusing on aesthetic aspects [32, 42], and includes focus on faces, as well as the adoption of GIST scene descriptors [43], which involve applying a set of oriented band-pass filters. Some of these features are explained below. For more complete information, refer to [1].*

*The* ***use of light*** *is a fundamental property of image aesthetics. Underexposed or overexposed pictures are usually considered to be of poor quality. In the HSV color space, the lightness of an image is measured as the average pixel value of the V channel.* ***HSV statistics****, specifically the mean of the S channel and the standard deviation of the S and V channels, are also analyzed.* ***Emotion-based*** *Saturation and Brightness directly influence pleasure, arousal, and dominance. The equations used for computing these are as follows: Pleasure = 0.69V + 0.22S, Arousal = -0.31V + 0.60S, and Dominance = 0.76V + 0.32S.* ***Colorfulness*** *allows us to distinguish between multi-colored images and those that are monochromatic, sepia, or simply low in contrast. It is measured using the Earth Mover’s Distance (EMD) between the image's histogram and a flat histogram, which represents a uniform color distribution. This measurement follows the algorithm suggested by Datta et al. [42].* ***Color naming*** *is a technique often used by photographers to showcase their personal style. Following [1], we consider the following 11 color names: black, blue, brown, grey, green, orange, pink, purple, red, white, and yellow.****”***

**Line 187: what is the usage of MDP here?**

**Response:**

**In this part, we have mentioned that MDP is the underlying reinforcement learning model and mentioned its main components.**

**Line 191: should be capital.**

**Response: Changes applied in the revised version.**

**Line 202: why do you use this algorithm?**

**Response: To apply this comment line 216 Revised as follows:**

**Line 216:** *“For this purpose, the deep deterministic policy gradient (DDPG) algorithm has been employed to generate recommendations. DDPG is well-suited for reinforcement learning tasks that involve continuous action spaces and complex, high-dimensional state spaces, and it can also simultaneously reduce redundant computation. These characteristics make it ideal for recommender systems. DDPG is an actor-critic method that merges the strategies of Q-learning and policy gradients, consisting of two models: the Actor network and the Critic network. ”*

**In the following, more explanations are given for the reason for using DDPG:**

One of the drawbacks of most reinforcement learning methods is that it cannot handle large and dynamic action space scenario, like recommender systems. Deep Deterministic Policy Gradient (DDPG) is designed for reinforcement learning tasks, particularly those involving continuous action spaces and complex, high-dimensional state spaces, while can also reduce redundant computation simultaneously. Here are a few reasons why DDPG might be considered useful for recommender systems:

1. Continuous Action Spaces: DDPG is well-suited for problems with continuous action spaces, where traditional recommender systems often involve discrete actions (e.g., recommending one item out of a fixed set). In some advanced recommender systems, actions can be considered as continuous parameters, such as the weights assigned to different features or items in a personalized recommendation.
2. Personalization: DDPG can be used to create highly personalized recommendation strategies. It learns an optimal policy for recommending items or content to individual users by taking into account user preferences, historical interactions, and contextual information, which can result in more accurate and relevant recommendations.
3. Exploration-Exploitation Trade-off: Recommender systems often face the exploration-exploitation trade-off. DDPG can help balance this trade-off by allowing for controlled exploration of recommendation strategies. It can adapt the recommendations based on user feedback and dynamically explore new items or content to better serve user interests.
4. Dynamic Environments: Some recommender systems operate in dynamic environments where user preferences and item availability change over time. DDPG's ability to adapt its policy in response to changing conditions can be advantageous in such scenarios.
5. Modeling User Behavior: DDPG can learn complex models of user behavior and preferences from historical interaction data. By using deep neural networks, it can capture intricate patterns and relationships in user-item interactions, leading to more accurate recommendations.

**Therefore, according to the advantages stated for DDPG, we have used this algorithm in the proposed method.**

**Line 207: where is the closed prantese?**

**Response: Changes applied in the revised version.**

**Line 221: what is the mechanism for this update?**

**Response: To answer this question, the following explanation has been added to the article.**

**Line 239:** *“In the Deep Deterministic Policy Gradient (DDPG) algorithm, both the actor and critic networks have their respective loss functions, which guide the learning process. These loss functions are central to the training and updating mechanism. The critic is updated, with the objective of minimizing the difference between the predicted Q-values and the target Q-values. The loss function for the critic is typically the Mean Squared Error (MSE) between these two values:*

$L\left( \theta^{Q} \right)= \mathbb{E}_{(s,a,r,s^{'}\mathcal{)\sim D}}\left[ \left( Q\left( s,a | \theta^{Q} \right)-y \right)^{2} \right]$	*(2)*

*Where* $\theta^{Q}$ *are the parameters of the critic network, and* $y$ *is the target value, computed as* $y=r+\gamma Q^{'}(s^{'},\mu^{'}(s^{'}|\theta^{\mu^{'}})|\theta^{Q^{'}})$*.* $\gamma$ *is the discount factor.* $Q^{'}(s^{'},\mu^{'}(s^{'}|\theta^{\mu^{'}})|\theta^{Q^{'}})$ *is the target Q-value for the next state, using the target critic network's parameters* $\theta^{Q^{'}}$ *and the target actor network* $\mu^{'}$ *for action selection.* $\mathcal{D}$ *is a replay buffer containing tuples of (state, action, reward, next state).*

*The actor's policy is updated using policy gradient methods. The objective is to maximize the Q-value output by the critic for the actions chosen by the actor. However, in practice, we perform gradient ascent on the expected Q-values predicted by the critic for the current policy. The loss function for the actor is essentially the negative of the expected Q-value:*

$L\left( \theta^{\mu} \right)= \mathbb{-E}_{s\sim\mathcal{D}}\left[ Q\left( s,\mu(s | \theta^{\mu} \right)|\theta^{Q}) \right]$	*(3)*

*Where* $\theta^{\mu}$ *are the parameters of the actor network.* $\mu(s,\theta^{\mu})$ *is the current policy's action given state* $s$*. The expectation is approximated by sampling from the replay buffer.*

*For each iteration (or batch of experiences), the critic's parameters* $\theta^{Q}$*are updated by minimizing the critic loss. This typically involves calculating gradients of* $L(\theta^{Q})$ *with respect to* $\theta^{Q}$ *and performing a step of gradient descent.The actor's parameters* $\theta^{\mu}$ *are updated by performing gradient ascent on the expected Q-values. The gradients required for the update are derived from the critic's Q-value output with respect to the actor's parameters. This is where the dependency of the actor on the critic's assessment comes into play, guiding the actor to choose actions that the critic values more. These updates are typically applied in a loop, where a batch of experiences is sampled from the replay buffer, the critic is updated, and then the actor is updated. Target networks for both the actor and the critic are also maintained, with their weights slowly tracked to the main networks' weights to stabilize learning. This process repeats for many episodes and steps, gradually improving both the actor's policy and the critic's value estimation.”*

**Line 243: referring to the equation should be the same format in al part the paper. Please look at lines 233 and 234.**

**Response: Changes applied in the revised version.**

**Line 263: why to map to zero to one interval and not -1 to 1?**

**Response:**

**Mapping data to the [0, 1] interval (also known as normalization) or the [-1, 1] interval (known as centering and scaling or standardization) depends on the specific context and goals of data preprocessing. Both approaches have their use cases. Mapping to [0, 1] Interval (Normalization) is commonly used when we want to scale data to a fixed range [0, 1], which can be useful in various machine learning algorithms, especially those that are sensitive to the scale of the input features, like neural networks and support vector machines. It ensures that all values are within a standardized range, which can prevent some algorithms from converging slowly or not converging at all. It preserves the relative differences between data points, maintaining the distribution shape. Therefor we have used this approach.**

**Line 270: Explain the mapping between this part and first part of Table1?**

**Response: To apply this comment following changes have been applied:**

**Line 309:** *“In this study, we used four datasets. 1) The Flickr Style dataset[45] for recognizing image style. The original dataset consisted of 80,000 images with style labels, and was classified into 20 labels. We could not collect all the images because some were unlinked from Flickr. In this study, 15 style labels are used, and we have 2900 images for each style. Fig. 3 shows seven sample images from this dataset, depicting styles such as serene, melancholy, ethereal, noir, vintage, romantic, and horror. We use this dataset to train a model to extract style features as defined in Table 1.*

*2) The data set introduced in [33] was utilized for emotion detection in images. It encompasses eight emotions as defined in Table 2, which include amusement, anger, awe, contentment, disgust, excitement, fear, and sadness. The version of the dataset we used contains fewer images than the original due to some images being lost. Table 2 lists the eight emotions and the corresponding number of images for each, while Fig 4 shows sample images with their labeled emotions.*

*3) PsychoFlickr dataset is used for personality analysis [38]. A collection of 60,000 images tagged as favorites by 300 Pro Flickr users (200 randomly selected favorites per user). This dataset is used to train model for extract Personality features define in Table 1.”*

**Line 283: if the tags of users on an image have meaningful difference, then how the situation is handled?**

**Response:**

**What we mean by tagging the image as favorites is that the user has liked the image. We have not considered tags or captions for images. An image is either liked by the user or not.**

**Line 314: Why this theory?**

**Response:**

**The Big Five (Five Factor Model) is widely accepted, reliable, and valid for assessing personality traits. It provides a comprehensive and relatively simple framework for understanding personality. This can be used when we want a general, broad assessment of personality traits that can be easily compared across individuals or populations. It's commonly used in research and applied settings. Therefore we have used this model for personality analysis in our work.**

**Line 328: are the difference between these two methods considerable?**

**Response:**

**We have investigated both models VGG16 and VGG19 to see which of them gives a better performance for personality analysis.**

**Line 339: more explanations are needed to justify to compare these methods! Is the comparison reasonable? What is the supporting logic?**

**Response: In this study, we focused on image-based recommendations and compared our method with others that also concentrated on image recommendation and didn’t consider any additional information. To this end, we compared our method with several that used the same dataset as ours. Additionally, for further investigation, we made comparisons with [6] and [44], which employ different datasets. We implemented each of the compared methods on our desired dataset to ensure a logical comparison. We compared our method with [25] due to their shared emphasis on using style as a feature for recommendation. Although these methods tackle the problem differently, their comparison is significant as it provides insights into the effectiveness of our method. The proposed method allows for dynamic user interaction with the recommendation system and offers various components to enhance the system.**

**In response to the comment, we have added an explanation in the revised manuscript, which is highlighted in blue for clarity.**

**Line 376:** *“In this section, we investigate the effectiveness of the proposed method for social image recommendation system. For this purpose, we have reviewed related works and compared the proposed method with some representative baseline methods. We focused on image-based recommendations and compared our method with others that also centered on image recommendation and do not consider any additional information. To this end, we compared our method with several that used the same dataset as ours. Additionally, for further investigation, we made comparisons with [6] and [46], which employ different datasets. We implemented each of the compared methods on our desired dataset to ensure a logical comparison.*

*We compared our method with [25] due to their shared emphasis on using style as a feature for recommendation. Although these methods tackle the problem differently, their comparison is significant as it provides insights into the effectiveness of our method. The proposed method allows for dynamic user interaction with the recommendation system and offers various components to enhance the system. The experimental results have been compared with the results obtained from the following methods:*

*(i) FGM, this method uses visual features and then applies FGM for simultaneous feature selection and classification [7].*

*(ii) LASSO, uses visual features and then LASSO regressor [1].*

*(iii) DeepFeatNN, Deep Features are extracted to learn user preferences. This method generates a user feature vector using averages all the image features liked by the user and then recommends an image that is more similar to the user's vector [6].*

*(ix) VOFeatFGMPFIS [24], this method use Visual and Object Features. For object features, VGGnet trained on 1000 object from ImageNet tag set [47] was used.*

*(x) Sulthana el al. [46], this method uses CNN features, applies dimensionality reduction, and employs cosine similarity for image recommendation.*

*(xi) VisualFeat.FTFMCT. FGM, This method uses style and a visual feature vector and then applies FGM for simultaneous feature selection and classification [25].”*

**Line 396: this interaction may lead to increase time complexity of the algorithm. Time and space complexity analysis of the method is required.**

**Response: To answer this comment, the following explanation has been added in the revised manuscript.**

**Line 525:**

“*In Table 5, time consumption of the compared methods are shown. We consider only training time for recommendation model and no consider training time for feature extractor models in proposed method.*

***Table 5: Comparison of running times (in seconds) of different methods.***

|  | Training time(s) | Test time(s) |
| --- | --- | --- |
| FGM(Guntuku et al.2016) | 1.32 | 0.003 |
| Lasso(Lovato et al. 2014) | 1.74 | 0.005 |
| Sulthana et al.2020 | 1794 | 0.028 |
| Ahmadkhani et al.2021 | 62 | 0.023 |
| Ahmadkhani et al.2022 | 2 | 0.01 |
| Proposed method | 2323 | 0.05 |

*In terms of training time, our method incurs the highest time investment compared to the others. This is due to the additional steps involved in our approach, including the training of a deep learning network. It should be noted that the proposed method interacts with the user and the training time depends on the number of episodes (The number of episodes to calculate the time presented in Table 5, is 100.). In addition, in the proposed method, without having training data, we can start learning the user's interests with just one image, and gradually learn the interests of the user. Therefore, according to the advantages stated for it, although the training time for the proposed method is high, it still has an advantage.*

*To calculate the space complexity, we must consider Model Memory and Experience Replay Buffer. For Model Memory, our proposed method maintains two neural networks: the actor (policy) network and the critic (value) network. The space complexity depends on the network architectures and the number of parameters in each network. DDPG uses an experience replay buffer to store past experiences for training. The space complexity of the replay buffer depends on its capacity, which is typically set to hold a fixed number of experiences (e.g., M experiences). The space complexity is O(M). Additionally, we have proposed considering a memory of the recommendation history. This memory keeps all the images recommended to the user, both those that are liked by the user and those that are not, which is typically set to hold a fixed number of experiences (e.g., Z experiences). The space complexity is O(Z).*

*Although the time complexity of the proposed method is more than other methods, it was noted that in addition to improving the recommendations compared to other methods, the proposed method is an interactive method through the use of deep reinforcement learning and is able to interact with the user from the beginning, and gradually improve. In other methods, we must have training data so that the model can be trained with it and then give recommendations to the user based on the trained model. Therefore, the proposed method can be more suitable for real-world applications.*

*To assess the empirical performance of our algorithm and complement the theoretical time complexity analysis, we executed our code on the Google Colab platform. The environment used for our tests included a runtime with GPU. The Colab notebooks were configured with a Python environment, and the code was executed using the pre-installed libraries available on Colab at the time of our experiments. It is important to note that while Google Colab environments are standardized in terms of software, the exact hardware specifications can vary and are abstracted from the user. Therefore, the reported execution times should be cofnsidered as an approximation.”*

**Line 401: the main contribution should be expressed clearly. Is the optimality of the method is new? How to prove that it is optimal? Is the strategy new?**

**Response: To respond to this comment, we revised the manuscript as follow:**

**Line 53:** *“For this study the main contributions can be summarized as follows:*

*1) Propose an image recommender system in a new deep reinforcement learning framework*

*2) Introducing a new method for state representation.*

*3) Introducing three components (style, Emotion, personality) that can be useful in images recommendation to create user's characteristics vector and investigate its effect on the recommended images on each user.* *”*

**Line 557:**

*“Due to encounter with the huge volume and variety of information, we need a system that can automatically identify and cater to the user's interests. The remarkable point about previous methods is that they often view the recommendation process as static, assuming that a user's underlying preferences remain unchanged. In reality, personalizing image recommendations according to individual user preferences is a complex task, as their preferences evolve over time. This evolution necessitates dynamic interaction between the system and the user, indicating the need for a dynamic recommendation process. Additionally, when limited data is available, it becomes difficult for a model to make accurate recommendations, an issue commonly referred to as the cold start problem. In this paper, we propose a dynamic image recommender system using a deep reinforcement learning framework and introduce a new method for state representation. This framework is novel for social image recommendations. Furthermore, we employ a set of newly extracted features, such as emotion, style, and personality, to enhance efficiency. In Section 4, the results of our tests demonstrate how the use of these components improves outcomes. Overall, the results indicate that our proposed method significantly outperforms some related works in personalized image recommendation.”*

**Comment- The main contribution(s) of the paper should be expressed clearly.**

**Response: To respond to this comment, we revised the manuscript as follow:**

**Line 53:** *“For this study the main contributions can be summarized as follows:*

*1) Propose an image recommender system in a new deep reinforcement learning framework*

*2) Introducing a new method for state representation.*

*3) Introducing three components (style, Emotion, personality) that can be useful in images recommendation to create user's characteristics vector and investigate its effect on the recommended images on each user.* *”*

**Line 557:**

*“Due to encounter with the huge volume and variety of information, we need a system that can automatically identify and cater to the user's interests. The remarkable point about previous methods is that they often view the recommendation process as static, assuming that a user's underlying preferences remain unchanged. In reality, personalizing image recommendations according to individual user preferences is a complex task, as their preferences evolve over time. This evolution necessitates dynamic interaction between the system and the user, indicating the need for a dynamic recommendation process. Additionally, when limited data is available, it becomes difficult for a model to make accurate recommendations, an issue commonly referred to as the cold start problem. In this paper, we propose a dynamic image recommender system using a deep reinforcement learning framework and introduce a new method for state representation. This framework is novel for social image recommendations. Furthermore, we employ a set of newly extracted features, such as emotion, style, and personality, to enhance efficiency. In Section 4, the results of our tests demonstrate how the use of these components improves outcomes. Overall, the results indicate that our proposed method significantly outperforms some related works in personalized image recommendation.”*

**Comment- Complexity analysis of the proposed method may be reported at least experimentally.**

**Response: To answer this comment, the following explanation has been added in the revised manuscript.**

**Line 525:**

“*In Table 5, time consumption of the compared methods are shown. We consider only training time for recommendation model and no consider training time for feature extractor models in proposed method.*

***Table 5: Comparison of running times (in seconds) of different methods.***

|  | Training time(s) | Test time(s) |
| --- | --- | --- |
| FGM(Guntuku et al.2016) | 1.32 | 0.003 |
| Lasso(Lovato et al. 2014) | 1.74 | 0.005 |
| Sulthana et al.2020 | 1794 | 0.028 |
| Ahmadkhani et al.2021 | 62 | 0.023 |
| Ahmadkhani et al.2022 | 2 | 0.01 |
| Proposed method | 2323 | 0.05 |

*In terms of training time, our method incurs the highest time investment compared to the others. This is due to the additional steps involved in our approach, including the training of a deep learning network. It should be noted that the proposed method interacts with the user and the training time depends on the number of episodes (The number of episodes to calculate the time presented in Table 5, is 100.). In addition, in the proposed method, without having training data, we can start learning the user's interests with just one image, and gradually learn the interests of the user. Therefore, according to the advantages stated for it, although the training time for the proposed method is high, it still has an advantage.*

*To calculate the space complexity, we must consider Model Memory and Experience Replay Buffer. For Model Memory, our proposed method maintains two neural networks: the actor (policy) network and the critic (value) network. The space complexity depends on the network architectures and the number of parameters in each network. DDPG uses an experience replay buffer to store past experiences for training. The space complexity of the replay buffer depends on its capacity, which is typically set to hold a fixed number of experiences (e.g., M experiences). The space complexity is O(M). Additionally, we have proposed considering a memory of the recommendation history. This memory keeps all the images recommended to the user, both those that are liked by the user and those that are not, which is typically set to hold a fixed number of experiences (e.g., Z experiences). The space complexity is O(Z).*

*Although the complexity of the proposed method is more than other methods, it was noted that in addition to improving the recommendations compared to other methods, the proposed method is an interactive method through the use of deep reinforcement learning and is able to interact with the user from the beginning, and gradually improve. In other methods, we must have training data so that the model can be trained with it and then give recommendations to the user based on the trained model. Therefore, the proposed method can be more suitable for real-world applications.*

*To assess the empirical performance of our algorithm and complement the theoretical time complexity analysis, we executed our code on the Google Colab platform. The environment used for our tests included a runtime with GPU. The Colab notebooks were configured with a Python environment, and the code was executed using the pre-installed libraries available on Colab at the time of our experiments. It is important to note that while Google Colab environments are standardized in terms of software, the exact hardware specifications can vary and are abstracted from the user. Therefore, the reported execution times should be considered as an approximation.”*

**Comment- Please add some recent references (I mean after 2022) to the paper.**

**Response: To apply this comment references with number [8], [18], [19], [20] and [21] have been added to the revised paper.**

**Line 62:** *“Recommender systems are one of the topics of researchers' attention, which are used in different fields [18-22].”*

**Line 31:** *“Reinforcement learning is increasingly used for its ability to tackle complex problems involving dynamic modeling and long-term planning [8].”*

**Comment- You use many subjects without any justification. If it is possible, please mention the reasons for your decisions where you made to build the proposed method.**

**Response: In light of your suggestions, we have now conducted a comprehensive ablation study to investigate the robustness and effectiveness of the various components of our algorithm. The details of this study are outlined below:**

**Line 499:**

*“*In this paper we proposed a dynamic Image recommender system using deep reinforcement learning frame work by employing a set of new extracted features includes emotion, style, and personality features. We claimed that using these components in the proposed framework improves the results. Therefore, the ablation study was designed to systematically evaluate the contribution of each component of our proposed framework. By considering one component at a time, we were able to assess its impact on the overall performance. This study has provided valuable insights into how each element contributes to the efficacy of our method. To this end, we considered three approaches:

1. In the first approach, we use visual and style features within the proposed framework to investigate the effect of style features on recommendations (This is shown as (***VS***) in the Fig 12 and 13).
2. In the second approach, we apply visual and emotion features to examine the impact of emotion features (This is shown as (***VE***) in the Fig 12 and 13).
3. In the third approach, we use visual and personality features within the proposed framework to study the influence of personality features on recommendations (This is shown as (***VP***) in the Fig 12 and 13).

We calculate Recall@K and Precision@K for all users using Eq. (10) and (11) for each approach. The results are shown in Fig. 12. The top row displays Recall@K for the three approaches, while the bottom row presents Precision@K. The results underscore the importance of these components within our method. These results indicate that incorporating each of these three components into the proposed framework improves performance. The ablation study outcomes strongly support our initial hypotheses and assertions about our proposed framework. Additionally, Figure 13 compares the effectiveness of each of these three approaches with the proposed method, which utilizes a combination of all three components within a deep reinforcement learning framework.

***Fig. 11: shows the results for three approaches: The top row displays Recall@K, and the bottom row shows Precision@K for different k values.***

***Fig. 12 the comparison between the three approaches and the proposed method***

***”***

**Reviewer #4:**

**Reviewer #4:** Today, due to the expansion of the Internet and social networks, people are faced with a huge amount of dynamic information. Recommender systems have emerged in order to improve the problem of the phenomenon of information abundance by analyzing the background of the user's behavior and extracting the user's interests and preferences. Most of the proposed methods for social image recommender systems since now use a non-dynamic strategy, this means they cannot adopt the method by changing user preferences. In this paper, authors have proposed a dynamic Image recommender system using deep reinforcement learning frame work by employing a set of new extracted features such as emotion, style, and personality features. In the proposed method, they overcome the change of state representation definition in reinforcement learning by introducing a new method for state representation. The experimental results show that our proposed method compared to some related works significantly improve Recall@k and Precision@k for personalized image recommendation. There are some novel contributions of this work, author must address my following queries.

**Comment- Author must proof read the manuscript to fix grammar errors.**

**Response: The language of the manuscript was reviewed and grammatical errors were corrected.**

**Comment- Author must present the run-time analysis of proposed research.**

**Response: To answer this comment, the following explanation has been added in the revised manuscript.**

**Line 525:**

“*In Table 5, time consumption of the compared methods are shown. We consider only training time for recommendation model and no consider training time for feature extractor models in proposed method.*

***Table 5: Comparison of running times (in seconds) of different methods.***

|  | Training time(s) | Test time(s) |
| --- | --- | --- |
| FGM(Guntuku et al.2016) | 1.32 | 0.003 |
| Lasso(Lovato et al. 2014) | 1.74 | 0.005 |
| Sulthana et al.2020 | 1794 | 0.028 |
| Ahmadkhani et al.2021 | 62 | 0.023 |
| Ahmadkhani et al.2022 | 2 | 0.01 |
| Proposed method | 2323 | 0.05 |

*In terms of training time, our method incurs the highest time investment compared to the others. This is due to the additional steps involved in our approach, including the training of a deep learning network. It should be noted that the proposed method interacts with the user and the training time depends on the number of episodes (The number of episodes to calculate the time presented in Table 5, is 100.). In addition, in the proposed method, without having training data, we can start learning the user's interests with just one image, and gradually learn the interests of the user. Therefore, according to the advantages stated for it, although the training time for the proposed method is high, it still has an advantage.*

*To calculate the space complexity, we must consider Model Memory and Experience Replay Buffer. For Model Memory, our proposed method maintains two neural networks: the actor (policy) network and the critic (value) network. The space complexity depends on the network architectures and the number of parameters in each network. DDPG uses an experience replay buffer to store past experiences for training. The space complexity of the replay buffer depends on its capacity, which is typically set to hold a fixed number of experiences (e.g., M experiences). The space complexity is O(M). Additionally, we have proposed considering a memory of the recommendation history. This memory keeps all the images recommended to the user, both those that are liked by the user and those that are not, which is typically set to hold a fixed number of experiences (e.g., Z experiences). The space complexity is O(Z).*

*Although the complexity of the proposed method is more than other methods, it was noted that in addition to improving the recommendations compared to other methods, the proposed method is an interactive method through the use of deep reinforcement learning and is able to interact with the user from the beginning, and gradually improve. In other methods, we must have training data so that the model can be trained with it and then give recommendations to the user based on the trained model. Therefore, the proposed method can be more suitable for real-world applications.*

*To assess the empirical performance of our algorithm and complement the theoretical time complexity analysis, we executed our code on the Google Colab platform. The environment used for our tests included a runtime with GPU. The Colab notebooks were configured with a Python environment, and the code was executed using the pre-installed libraries available on Colab at the time of our experiments. It is important to note that while Google Colab environments are standardized in terms of software, the exact hardware specifications can vary and are abstracted from the user. Therefore, the reported execution times should be considered as an approximation.”*

**Comment- The sample size use in this search model is quite small, I will suggest the authors to evaluate the proposed research on a large-scale deep learning dataset or otherwise a clear justification is required about this.**

**Response: In response to the comment, we have expanded the dataset to include 40 users. The results for the two best methods are depicted in Figure 11. These results illustrate that, even with the increased number of users, the proposed method retains its superiority. The revised manuscript has been updated accordingly, as follows:**

**Line 491:**

*“Modeling user preferences is a user-centric problem, where the number of images each user likes may be more significant than the total number of users. With this in mind, we chose a set of 200 images per user, in line with the numbers reported in [48] and [49]. Furthermore, we structured our dataset following the method used in [7], which includes 200 images for each of the 20 users. To validate the results, we expanded the dataset to include 40 users. The results for the two best methods are depicted in Figure 11. As Figure 11 illustrates, even with the increased number of users, the superiority of the proposed method remains evident.”*

**Fig. 11 Recall@k and Precision@k for different k values**

**Comment- The comparison with the baseline deep learning research models is missing.**

**Response: To answer this comment, we investigate the approach proposed for recommendation in [46] and compare with our proposed method. In this study, the authors delve into the investigation, execution, and evaluation of employing features derived from CNN alongside various dimensionality reduction techniques and similarity computation for image recommender system.**

**Line 396:** *“(x) Sulthana el al. [46], this method uses CNN features, applies dimensionality reduction, and employs cosine similarity for image recommendation.*”

**Line 454:** *“We calculate the recall@K and Precision@K on all users using Eqs. (2) and (3). Fig 6 and 7 show the results, we can see that the proposed method outperformed baselines, demonstrating our proposed method's effectiveness. Recall@K is used to calculate the proportion of relevant items in the top-K. Precision@K calculates the proportion of relevant top-K items (liked by the user). Also, in Fig 8 and 9, we have calculated NDCG@k and F1@K for all users and plotted its changes for different k, and these comparison criteria confirm the superiority of the proposed method.”*

**Fig. 6 Recall@k for different k values**

**Fig. 7 Precision@k for different k values**

**Fig. 8 NDCG@k for different k values**

**Fig. 9 F1@k for different k values**

**Comment-** **The contributions of proposed work are not clear, author must explicitly state the contributions that how each of the claim has been achieved.**

**Response: To respond to this comment, we revised the manuscript as follow:**

**Line 53:** *“For this study the main contributions can be summarized as follows:*

*1) Propose an image recommender system in a new deep reinforcement learning framework*

*2) Introducing a new method for state representation.*

*3) Introducing three components (style, Emotion, personality) that can be useful in images recommendation to create user's characteristics vector and investigate its effect on the recommended images on each user.* *”*

**Line 557:**

*“Due to encounter with the huge volume and variety of information, we need a system that can automatically identify and cater to the user's interests. The remarkable point about previous methods is that they often view the recommendation process as static, assuming that a user's underlying preferences remain unchanged. In reality, personalizing image recommendations according to individual user preferences is a complex task, as their preferences evolve over time. This evolution necessitates dynamic interaction between the system and the user, indicating the need for a dynamic recommendation process. Additionally, when limited data is available, it becomes difficult for a model to make accurate recommendations, an issue commonly referred to as the cold start problem. In this paper, we propose a dynamic image recommender system using a deep reinforcement learning framework and introduce a new method for state representation. This framework is novel for social image recommendations. Furthermore, we employ a set of newly extracted features, such as emotion, style, and personality, to enhance efficiency. In Section 4, the results of our tests demonstrate how the use of these components improves outcomes. Overall, the results indicate that our proposed method significantly outperforms some related works in personalized image recommendation.”*

**Comment-** **Author are suggested to cite and discuss**

• Nimrah, S., Saifullah, S.: Context-free word importance scores for attacking neural networks. Journal of Computational and Cognitive Engineering.1 (4), 187–192 (2022). <https://doi.org/10.47852/bonviewJCCE2202406>

• Ma, Y., et al.: Deep learning framework for multi-round service bundle recommendation in iterative mashup development. CAAI Trans. Intell. Technol. 1– 17 (2022). <https://doi.org/10.1049/cit2.12135>

• Fang, Q., et al.: Target-driven visual navigation in indoor scenes using reinforcement learning and imitation learning. CAAI Trans. Intell. Technol. 7(2), 167– 176 (2022). <https://doi.org/10.1049/cit2.12043>

**Response: Thank you for suggesting these works to study. Items 2, and 3 have been added as references [8], and [20] respectively. Item 1 was also studied and although the work is very valuable, it was not added due to lack of relevance with our work.**

**The paragraph to which the reference [8] is added:**

**Line 31:** *“Reinforcement learning is increasingly used for its ability to tackle complex problems involving dynamic modeling and long-term planning [8].”*

**The paragraph to which the reference [20] is added:**

**Line 62:** *“Recommender systems are one of the topics of researchers' attention, which are used in different fields [18-22]. In this section, we divide recommender systems into two categories: Non RL or traditional recommendation systems and RL based recommendation systems.”*

**Comment- Results must be compared with state-of-the-art published values.**

**Response: To answer this comment, we investigate the approach proposed for recommendation in [46] and compare with our proposed method. In this study, the authors delve into the investigation, execution, and evaluation of employing features derived from CNN alongside various dimensionality reduction techniques and similarity computation for image recommender system.**

**Line 396:** *“(x) Sulthana el al. [46], this method uses CNN features, applies dimensionality reduction, and employs cosine similarity for image recommendation.*”

**Line 454:** *“We calculate the recall@K and Precision@K on all users using Eqs. (2) and (3). Fig 6 and 7 show the results, we can see that the proposed method outperformed baselines, demonstrating our proposed method's effectiveness. Recall@K is used to calculate the proportion of relevant items in the top-K. Precision@K calculates the proportion of relevant top-K items (liked by the user). Also, in Fig 8 and 9, we have calculated NDCG@k and F1@K for all users and plotted its changes for different k, and these comparison criteria confirm the superiority of the proposed method.”*

**Fig. 6 Recall@k for different k values**

**Fig. 7 Precision@k for different k values**

**Fig. 8 NDCG@k for different k values**

**Fig. 9 F1@k for different k values**

**Comment- There are many blurry figures that must be redrawn.**

**Response: we have carefully reviewed all the figures in the manuscript. We have identified the figures that were not as clear as required and redrawing Blurry Figures. Figures 1, 2, and 5 have been meticulously redrawn to ensure higher resolution and clearer visual details.**

**Fig. 1 Workflow of the proposed method (The images shown as input and output in this workflow are from reference [1]).**

**Fig. 2 State representation using self-attention**

**Fig. 4. Sample images of eight different categories of emotions (These images are from the dataset introduced in reference** [**33]). Top row: four positive emotions and bottom row: four negative emotions.**

**Comment-** **Optimization details are not clear in the current version.**

**Response: there are parameters in the proposed method, must be adjusted which is described in the revised manuscript as follow:**

**Line 446:**

*“In our experiments, we conduct 400 episodes using the proposed method. In each episode, 100 unique images are recommended to the user; we ensure that items already recommended in previous episodes are not repeated by removing them from the candidate set. Additionally, to make decisions based on the users' most recent interactions, we store up to 100 images (regardless of user liked or not) recommended in each episode, forming what we refer to as the 'recommendation history'. The discount rate (γ) is set at 0.3. For all reinforcement learning-based methods, we employ the Adam optimizer. The mini-batch size is configured at 10, and the initial learning rate is set to 0.001. We determine the optimal parameters, including the hidden layer sizes for both the actor and critic networks, through grid search.”*

**Additionally, the section on the proposed method has been rewritten in the revised manuscript to provide a more detailed description of the method:**

**Line 190:**

*“In this research, we construct a user recommendation model for each individual user, denoted as user i, based on a collection of M images that the user has marked as favorites. Imagining a scenario with N distinct users, represented as* $U=\left\{ u_{1}, u_{2},\ldots,u_{N} \right\}$*, and a corresponding set of M images* $I=\{I_{1},I_{2},\ldots,I_{M}\}$*, with* $I_{i}=\{x_{1}^{i},x_{2}^{i},\ldots,x_{k}^{i}\}$ *signifying the subset of images liked by user I, and* $e_{i}$ *expression is the feature vector representation of item* $x^{i}$*. Accordingly, a portion of each user's preferred images is allocated for the training set, while the remainder is utilized for testing purposes.*

*We model our proposed method in the reinforcement learning setting. An agent is a recommendation system in our context, which interacts with the environment (users) and receives rewards from the environment (feedback from users). Rewards serve as an index of whether the course of action the agent is taking is right or wrong. The agent is constantly in contact with the environment, and eventually learns to take the right action through the feedback received from the environment over a period of time. The underlying reinforcement learning model is the Markov Decision Process (MDP) includes a sequence of states, actions, and rewards. MDP is determined by five components as follows:*

***State space S****: Created by the environment, this represents the agent's situation at any given moment. In our work, the status s indicates the user's positive interaction history with the recommender, and we have proposed a state representation module to represent it (Section 3-2-s1).*

***Action space A****: An action a ∈ A includes possible reactions that the agent may exhibit in response to the current state. This could be a list of items recommended to the user in the current state.*

***Reward R****: This is the immediate feedback sent to the agent by the environment after each action is evaluated.*

***Transition probability P****: This denotes the probability of transitioning from state*$s_{t}$ *to state* $s_{t+1}$ *when the agent performs action*$a_{t}$*.*

***Discount factor γ****: It is a factor that measures the present value of long-term rewards. Its value is between 0 and 1. If γ = 0, the recommender ignores long-term rewards and considers only immediate ones. But, when γ = 1, the recommender considers long-term and immediate rewards equally important.*

*As previously mentioned, most work has focused solely on static recommendation processes using pre-trained models, which do not effectively simulate the dynamic interaction between users and their systems. Therefore, we propose a new social image recommender system within the deep reinforcement learning framework. For this purpose, the deep deterministic policy gradient (DDPG) algorithm has been employed to generate recommendations. DDPG is well-suited for reinforcement learning tasks that involve continuous action spaces and complex, high-dimensional state spaces, and it can also simultaneously reduce redundant computation. These characteristics make it ideal for recommender systems. DDPG is an actor-critic method that merges the strategies of Q-learning and policy gradients, consisting of two models: the Actor network and the Critic network.*

***The actor, or policy network,*** *takes the state of a user as input and generates an appropriate action. We determine the user's state vector at each step using the state representation module, which assesses the user's state based on their history of liked images (The proposed module for state representation is described in Section 3-2-1). The actor-network is composed of two ReLU layers, one Tanh layer, and incorporates a skip connection in the penultimate layer. This network receives the user state and outputs the action vector, which is then used to make recommendations to the user. Since there are numerous images to potentially recommend, we employ a scoring function based on the inner product, defined as follows, to select the most appropriate image for recommendation.*

${score}_{i}=e_{i}^{T}a$	*(1)*

*Where a is the output of Actor and* $e_{i}$ *expression is the vector representation of item* $x^{i}$*. After calculating the scores, the images are ranked based on the scores, and the image with the top rank is recommended.*

***The critic network****, is a Deep Q-Network design as a deep neural network parameterized*$Q(s,a)$*. The generated action vector and the user's state are given as input to the critic network to determine how good the generated action is. According to* $Q(s,a)$*, the Actor network's parameters are updated in order to improve the performance of action a, that is, to enhance* $Q(s,a)$*.*

*In the Deep Deterministic Policy Gradient (DDPG) algorithm, both the actor and critic networks have their respective loss functions, which guide the learning process. These loss functions are central to the training and updating mechanism. The critic is updated, with the objective of minimizing the difference between the predicted Q-values and the target Q-values. The loss function for the critic is typically the Mean Squared Error (MSE) between these two values:*

$L\left( \theta^{Q} \right)= \mathbb{E}_{(s,a,r,s^{'}\mathcal{)\sim D}}\left[ \left( Q\left( s,a | \theta^{Q} \right)-y \right)^{2} \right]$	*(2)*

*Where* $\theta^{Q}$ *are the parameters of the critic network, and* $y$ *is the target value, computed as* $y=r+\gamma Q^{'}(s^{'},\mu^{'}(s^{'}|\theta^{\mu^{'}})|\theta^{Q^{'}})$*.* $\gamma$ *is the discount factor.* $Q^{'}(s^{'},\mu^{'}(s^{'}|\theta^{\mu^{'}})|\theta^{Q^{'}})$ *is the target Q-value for the next state, using the target critic network's parameters* $\theta^{Q^{'}}$ *and the target actor network* $\mu^{'}$ *for action selection.* $\mathcal{D}$ *is a replay buffer containing tuples of (state, action, reward, next state).*

*The actor's policy is updated using policy gradient methods. The objective is to maximize the Q-value output by the critic for the actions chosen by the actor. However, in practice, we perform gradient ascent on the expected Q-values predicted by the critic for the current policy. The loss function for the actor is essentially the negative of the expected Q-value:*

$L\left( \theta^{\mu} \right)= \mathbb{-E}_{s\sim\mathcal{D}}\left[ Q\left( s,\mu(s | \theta^{\mu} \right)|\theta^{Q}) \right]$	*(3)*

*Where* $\theta^{\mu}$ *are the parameters of the actor network.* $\mu(s,\theta^{\mu})$ *is the current policy's action given state* $s$*. The expectation is approximated by sampling from the replay buffer.*

*For each iteration (or batch of experiences), the critic's parameters* $\theta^{Q}$*are updated by minimizing the critic loss. This typically involves calculating gradients of* $L(\theta^{Q})$ *with respect to* $\theta^{Q}$ *and performing a step of gradient descent.The actor's parameters* $\theta^{\mu}$ *are updated by performing gradient ascent on the expected Q-values. The gradients required for the update are derived from the critic's Q-value output with respect to the actor's parameters. This is where the dependency of the actor on the critic's assessment comes into play, guiding the actor to choose actions that the critic values more. These updates are typically applied in a loop, where a batch of experiences is sampled from the replay buffer, the critic is updated, and then the actor is updated. Target networks for both the actor and the critic are also maintained, with their weights slowly tracked to the main networks' weights to stabilize learning. This process repeats for many episodes and steps, gradually improving both the actor's policy and the critic's value estimation.*

*Given the vast number of images available for recommendation, we propose utilizing a memory of the user's recommendation history to aid the recommendation process with the help of the Action vector. This memory records all images recommended to the user, including those they liked and those they did not. We employ the Lasso algorithm to derive a vector of coefficients from the stored information. We then calculate the dot product between this coefficient vector (*$\beta$*) and the candidate images to select a list that represents the most suitable recommendations.*

${score}_{i\beta}=x_{j}\beta$	*(4)*

*After calculating the* ${score}_{j\beta}$ *for all* $x_{j}$*, we consider those with higher scores as better candidates for recommendation. We then compute the dot product of the list of selected images with the action vector obtained from the actor network to create recommendations as outlined in Eq (1).The reward function is defined by Eq (5). If a recommended image is liked by the user, the system receives a positive reward; conversely, if the image is not liked, the system receives a negative reward.*

$Reward\left( Image \right)= \left\{ \begin{matrix} \begin{aligned} 1 If the user likes the image \\ -1 If the user does not like the image \end{aligned} \\ \end{matrix} \right.$	*(5)*

*”*

**Comment- Mathematical model is not explained well.**

**Response: To answer this comment, we have added explanations in the revised manuscript, which is highlighted in blue for clarity. The proposed method section has been rewritten in the revised manuscript as follows:**

**Line 190:**

*“*In this research, we construct a user recommendation model for each individual user, denoted as user *i*, based on a collection of M images that the user has marked as favorites. Imagining a scenario with N distinct users, represented as $U=\left\{ u_{1}, u_{2},\ldots,u_{N} \right\}$, and a corresponding set of M images $I=\{I_{1},I_{2},\ldots,I_{M}\}$, with $I_{i}=\{x_{1}^{i},x_{2}^{i},\ldots,x_{k}^{i}\}$ signifying the subset of images liked by user *I*, and $e_{i}$ expression is the feature vector representation of item $x^{i}$. Accordingly, a portion of each user's preferred images is allocated for the training set, while the remainder is utilized for testing purposes.

We model our proposed method in the reinforcement learning setting. An agent is a recommendation system in our context, which interacts with the environment (users) and receives rewards from the environment (feedback from users). Rewards serve as an index of whether the course of action the agent is taking is right or wrong. The agent is constantly in contact with the environment, and eventually learns to take the right action through the feedback received from the environment over a period of time. The underlying reinforcement learning model is the Markov Decision Process (MDP) includes a sequence of states, actions, and rewards. MDP is determined by five components as follows:

**State space *S***: Created by the environment, this represents the agent's situation at any given moment. In our work, the status s indicates the user's positive interaction history with the recommender, and we have proposed a state representation module to represent it (Section 3-2-s1).

**Action space A**: An action a ∈ A includes possible reactions that the agent may exhibit in response to the current state. This could be a list of items recommended to the user in the current state.

**Reward R**: This is the immediate feedback sent to the agent by the environment after each action is evaluated.

**Transition probability P**: This denotes the probability of transitioning from state$s_{t}$ to state $s_{t+1}$ when the agent performs action$a_{t}$.

**Discount factor γ**: It is a factor that measures the present value of long-term rewards. Its value is between 0 and 1. If γ = 0, the recommender ignores long-term rewards and considers only immediate ones. But, when γ = 1, the recommender considers long-term and immediate rewards equally important.

As previously mentioned, most work has focused solely on static recommendation processes using pre-trained models, which do not effectively simulate the dynamic interaction between users and their systems. Therefore, we propose a new social image recommender system within the deep reinforcement learning framework. For this purpose, the deep deterministic policy gradient (DDPG) algorithm has been employed to generate recommendations. DDPG is well-suited for reinforcement learning tasks that involve continuous action spaces and complex, high-dimensional state spaces, and it can also simultaneously reduce redundant computation. These characteristics make it ideal for recommender systems. DDPG is an actor-critic method that merges the strategies of Q-learning and policy gradients, consisting of two models: the Actor network and the Critic network.

**The actor, or policy network,** takes the state of a user as input and generates an appropriate action. We determine the user's state vector at each step using the state representation module, which assesses the user's state based on their history of liked images (The proposed module for state representation is described in Section 3-2-1). The actor-network is composed of two ReLU layers, one Tanh layer, and incorporates a skip connection in the penultimate layer. This network receives the user state and outputs the action vector, which is then used to make recommendations to the user. Since there are numerous images to potentially recommend, we employ a scoring function based on the inner product, defined as follows, to select the most appropriate image for recommendation.

${score}_{i}=e_{i}^{T}a$	(1)

Where *a* is the output of Actor and $e_{i}$ expression is the vector representation of item $x^{i}$. After calculating the scores, the images are ranked based on the scores, and the image with the top rank is recommended.

**The critic network**, is a Deep Q-Network design as a deep neural network parameterized$Q(s,a)$. The generated action vector and the user's state are given as input to the critic network to determine how good the generated action is. According to $Q(s,a)$, the Actor network's parameters are updated in order to improve the performance of action *a*, that is, to enhance $Q(s,a)$.

In the Deep Deterministic Policy Gradient (DDPG) algorithm, both the actor and critic networks have their respective loss functions, which guide the learning process. These loss functions are central to the training and updating mechanism. The critic is updated, with the objective of minimizing the difference between the predicted Q-values and the target Q-values. The loss function for the critic is typically the Mean Squared Error (MSE) between these two values:

$L\left( \theta^{Q} \right)= \mathbb{E}_{(s,a,r,s^{'}\mathcal{)\sim D}}\left[ \left( Q\left( s,a | \theta^{Q} \right)-y \right)^{2} \right]$	(2)

Where $\theta^{Q}$ are the parameters of the critic network, and $y$ is the target value, computed as $y=r+\gamma Q^{'}(s^{'},\mu^{'}(s^{'}|\theta^{\mu^{'}})|\theta^{Q^{'}})$. $\gamma$ is the discount factor. $Q^{'}(s^{'},\mu^{'}(s^{'}|\theta^{\mu^{'}})|\theta^{Q^{'}})$ is the target Q-value for the next state, using the target critic network's parameters $\theta^{Q^{'}}$ and the target actor network $\mu^{'}$ for action selection. $\mathcal{D}$ is a replay buffer containing tuples of (state, action, reward, next state).

The actor's policy is updated using policy gradient methods. The objective is to maximize the Q-value output by the critic for the actions chosen by the actor. However, in practice, we perform gradient ascent on the expected Q-values predicted by the critic for the current policy. The loss function for the actor is essentially the negative of the expected Q-value:

$L\left( \theta^{\mu} \right)= \mathbb{-E}_{s\sim\mathcal{D}}\left[ Q\left( s,\mu(s | \theta^{\mu} \right)|\theta^{Q}) \right]$	(3)

Where $\theta^{\mu}$ are the parameters of the actor network. $\mu(s,\theta^{\mu})$ is the current policy's action given state $s$. The expectation is approximated by sampling from the replay buffer.

For each iteration (or batch of experiences), the critic's parameters $\theta^{Q}$are updated by minimizing the critic loss. This typically involves calculating gradients of $L(\theta^{Q})$ with respect to $\theta^{Q}$ and performing a step of gradient descent. The actor's parameters $\theta^{\mu}$ are updated by performing gradient ascent on the expected Q-values. The gradients required for the update are derived from the critic's Q-value output with respect to the actor's parameters. This is where the dependency of the actor on the critic's assessment comes into play, guiding the actor to choose actions that the critic values more. These updates are typically applied in a loop, where a batch of experiences is sampled from the replay buffer, the critic is updated, and then the actor is updated. Target networks for both the actor and the critic are also maintained, with their weights slowly tracked to the main networks' weights to stabilize learning. This process repeats for many episodes and steps, gradually improving both the actor's policy and the critic's value estimation.

Given the vast number of images available for recommendation, we propose utilizing a memory of the user's recommendation history to aid the recommendation process with the help of the Action vector. This memory records all images recommended to the user, including those they liked and those they did not. We employ the Lasso algorithm to derive a vector of coefficients from the stored information. We then calculate the dot product between this coefficient vector ($\beta$) and the candidate images to select a list that represents the most suitable recommendations.

${score}_{i\beta}=x_{j}\beta$	(4)

After calculating the ${score}_{j\beta}$ for all $x_{j}$, we consider those with higher scores as better candidates for recommendation. We then compute the dot product of the list of selected images with the action vector obtained from the actor network to create recommendations as outlined in Eq (1).The reward function is defined by Eq (5). If a recommended image is liked by the user, the system receives a positive reward; conversely, if the image is not liked, the system receives a negative reward.

$Reward\left( Image \right)= \left\{ \begin{matrix} \begin{aligned} 1 If the user likes the image \\ -1 If the user does not like the image \end{aligned} \\ \end{matrix} \right.$	(5)

*”*
