# Supplementary material for: A social image recommendation system based on deep reinforcement learning
Source: PLoS One. 2024 Apr 4;19(4):e0300059. doi: 10.1371/journal.pone.0300059 (PMC10994284; doi:10.1371/journal.pone.0300059)
Supplement: Attachment — Submitted filename: Response to Reviewers.docx [file pone.0300059.s005.docx]

**Overall Comment: Minor Revisions**

**Dear respectful editor and reviewers,**

**We would like to appreciate your comments to improve the paper quality. We tried our best to address all the comments. Hope that the revised paper satisfies the publications’ requirements.**

**Sincerely,**

**Corresponding author**

**Response to Reviewer Comment**

**1. The authors are advised to thoroughly review the manuscript as inconsistencies in font sizes have been observed in the text. A notable example of this inconsistency can be found in Lines 148-158.**

**Response:**

**Thank you for bringing this to our attention. We understand the importance of maintaining a consistent presentation throughout the manuscript, as it not only ensures clarity but also reflects the professional quality of the work.**

**We conducted a thorough review of the entire manuscript to identify and correct any such inconsistencies. We ensured that the font size remained uniform throughout the text, adhering to the journal's guidelines for manuscript formatting.**

**2. Please do not add arXiv preprint papers to the reference list, as they are not peer-reviewed. Replace them with other similar papers.**

**Response:**

**In light of your advice, we have reviewed our reference list and replaced the arXiv preprint citations with alternative sources. We have ensured that these replacements are from peer-reviewed journals or conferences, closely related to the subject matter of the removed references, and equally contribute to the context and support of our research findings. References 14, 29, and 30 have been changed.**

**3. In the related works section, authors should compare the differences and shortcomings of different works rather than simply listing and summarizing them.**

**Response: To response to this comment, we have revised the related work section as follows:**

**Line 69:**

*“Some studies have investigated feature extraction methods from images to understand user interests. Lovato et al. [1] considered the images tagged as favorites by a user and extracted handcrafted features from them such as HSV statistics, Use of light, and etc. Although these features can be useful, there is still a need to extract more features. Guntuku et al. [7], in addition to low-level features [1], extracted a set of high-level features such as Head and Upperbody recognition, Visual Clutter, tag, etc. They proposed a deep bimodal knowledge representation model that increased efficiency by using visual and tag features. The method presented in [23] extracts various features, applies feature selection to take the important features, and then uses fuzzy inference system for image recommendation. They assert that fuzzy logic, as a decision-making system, adeptly handles vague and inaccurate information. This makes it suitable for modeling the uncertain and ambiguous preferences of users in recommender systems. In these methods, image-level features and social information such as tags are utilized to enhance recommendations. In [24], introduction of style as a new component in social image recommendation is presented. This demonstrates that incorporating style significantly improves the results.*

*In some previous researches, image-level features, deep methods, and social information have been noticed to determine user preferences [25-27]. However, defining a new component related to user preferences can be useful.*

*Most traditional recommendation methods involve user-item interaction modeling with supervised learning such as classification, memory-based content filtering from user history, etc. These methods ignore the dependence during successive time steps. Unlike the supervised learning setting, where a guide tells you the right action, to better reflect the user-system interaction, it is widely agreed that the formulation of the problem as a sequential decision problem can be better [15]. Therefore, it can be solved by RL relies on the environment to discover the right action. In RL, the learning process is through interaction with an environment.*

*Shani et al. [9] used Markov decision processes (MDPs) for recommender systems to consider the long-term effect of recommendations and the expected value of each recommendation. Taghipour et al. [10] used Q-Learning to model web page recommendation. However, these model-based RL techniques [10] are inapplicable when there are many candidate items for recommendation, because updating the model requires a time-consuming dynamic programming step. Therefore, model-free RL techniques were preferred for use in recommender systems. These techniques can be divided into two categories: value-based [11, 12] and policy-based [14, 15].*

*In value-based approaches, for a given state, one must calculate the Q values of all available actions, then the action that has the maximum Q-value is selected as the best action. Therefore, when we face a very large action space, the approaches may become very inefficient.*

*The policy-based approaches generate a continuous parameter vector to represent an action [14, 15]. This vector can generate the recommendation and update the Q-value evaluator; so can overcome the inefficiency drawbacks.*

*As deep neural networks developed; deep RL emerged. Deep RL techniques have recently attracted the attention of recommender systems because they enable the use of RL in problems with large state and action spaces. Zheng et al. [11] applied deep Q-learning in news recommendations to effectively model the dynamic news features and user preferences. The Dueling Q-network is utilized in [11] to model the Q-value of a state-action pair. These value-based approaches require evaluating the Q-values of all actions under a specific state, which becomes highly inefficient when the number of actions is large. Zhao et al.[13] proposed List-wise recommendations based on Deep RL, which can be applied in scenarios with large and dynamic item space. In another study, Zhao et al. [28] used Deep RL for page-wise recommendation to learn the optimal recommendation strategies and optimizes a page of items simultaneously. However, there was another limitation concerning the user state, which did not explicitly and carefully model the interactions between users and items. Liu et al. [16] used an actor-critic RL approach to model the interactions between users and recommender systems for dynamic adaptation and long-term rewards. Huang et al. [29] considered the recommendation process as a Markov decision process and proposed a top-N model based on deep RL for long-term prediction, wherein recurrent neural network is used to simulate the interactions between the recommender system and users.”*

**4. The specific terms in the manuscript lack consistency. For instance, "deep reinforcement learning" is abbreviated as "DRL" in the Introduction, but variations such as "Deep RL" and "Deep Reinforcement Learning" persist in subsequent chapters.**

**Response:**

**We undertook a meticulous revision of the manuscript to standardize the terminology.**

**Specifically, we ensured that the term ' reinforcement learning' and its abbreviation 'RL' , and 'deep reinforcement learning' and its abbreviation 'Deep RL' were used appropriately throughout the document.**

**This revision extended to all terms and abbreviations within the manuscript, ensuring that each was used consistently and in accordance with the standard practices in our field.**

**5. The quality of the figures in this paper can be greatly improved. In the current version of this paper, some figures have small font sizes, which are hard to read, such as Fig. 1.**

**Response:**

**We acknowledge that the font sizes in some figures, particularly in Fig. 1, are indeed too small to be easily readable. We rectified this by resizing and enhancing the figures 1, 12 and 13 in the manuscript. This included increasing the font size to ensure that all text within the figures was clearly legible.**

**In addition, high resolution images have been attached to the manuscript as supplementary materials to make it possible zooming and reading well.**

**Line 122:**

**Fig. 1 Workflow of the proposed method (The images shown as input and output in this workflow are from reference [1]).**

**Line 520:**

**Fig. 12: shows the results for three approaches: The top row displays Recall@K, and the bottom row shows Precision@K for different k values.**

**Line 523:**

**Fig. 13 The comparison between the three approaches and the proposed method**

**6.** **The authors should explain the models trained deeply, showing more details of the experiments, such as the experiment running environment, including software and hardware configurations.**

**Response:**

**We have provided explanations and responses to this comment as follows:**

Line 404:

*“To evaluate image recommendation, we consider a dataset comprising 4,000 images from 20 Flickr users, as referenced in [1]. Each user has 200 images tagged as favorites. For comparison purposes, we split the dataset evenly into training and testing sets. Since we only have access to the images liked by users and lack those they did not like, we needed to create two sets of positive and negative samples for each user. Therefore, for positive samples we selected 100 images of 200 images “liked” by the user. To determine negative samples, we calculated the similarity between users and then selected 100 images of 200 images “liked” by each of ten users with the most distance from the user. For the test set the remaining images of users are used.*

*In our experiments, we conduct 400 episodes using the proposed method, with 100 images recommended to the user during each episode. We store these 100 images in the recommendation history, whether he likes them or not. At the beginning of each episode, we apply lasso regression on the data stored in the history of recommendations to determine the features that are most important to the user for liking the image. We use the vector obtained from Lasso to filter a large set of images that are candidates for recommendation to the user. Then we use the set of images obtained from this filter to apply Eq. (1) and obtain the score of each image. After calculating the scores with Eq. (1), the images are ranked based on the scores and the image with the highest score will be recommended. These repetitions and the use of the lasso filter help update the parameters of the actor and critic networks according to the user's interests and advance the system toward providing better recommendations. At the beginning of the system, we set the initial status with one image liked by the user, and at each step, the user's state is updated by the state representation module. This issue can solve the problem of cold start which is one of the problems of recommender systems because the system starts with the least possible information from the user and updates itself with the user's preferences at every step.”*

Line 448:

*“In our proposed method the actor-network is composed of two ReLU layers, one Tanh layer, and incorporates a skip connection in the penultimate layer. This network receives the user state and outputs the action vector, which is then used to make recommendations to the user. The critic-network is composed of three ReLU layers, and incorporates a skip connection in the penultimate layer. The dimension of the image embedding vector determines based on features extracted and is 107. In our experiments, we conduct 400 episodes using the proposed method. In each episode, 100 unique images are recommended to the user; we ensure that items already recommended in previous episodes are not repeated by removing them from the candidate set. Additionally, to make decisions based on the users' most recent interactions, we store up to 100 images (regardless of user liked or not) recommended in each episode, forming what we refer to as the 'recommendation history'. The discount rate (γ) is set at 0.3. For all RL-based methods, we employ the Adam optimizer. The mini-batch size is configured at 10, and the initial learning rate is set to 0.001. We determine the optimal parameters, including the hidden layer sizes for both the actor and critic networks, through grid search. In the implementation of our experiments, we utilized Google Colab, a cloud-based platform that provides an accessible and powerful computing environment. Depending on availability, Google Colab typically offers either the NVIDIA K80 or T4 GPUs. The K80 comes with 12GB of GPU memory, whereas the T4 is equipped with 16GB. The virtual machines provided by Google Colab are equipped with 12GB of RAM, ensuring efficient handling of large datasets and computationally intensive tasks. All our code was written in Python. We specifically used Python 3.10.12. Our models were developed and trained using TensorFlow. We employed TensorFlow version 2.12.0 , which provided the necessary tools and functionalities for our models.”*
