# Supplementary material for: A social image recommendation system based on deep reinforcement learning
Source: PLoS One. 2024 Apr 4;19(4):e0300059. doi: 10.1371/journal.pone.0300059 (PMC10994284; doi:10.1371/journal.pone.0300059)
Supplement: Attachment — Submitted filename: Response to Reviewers'.docx [file pone.0300059.s006.docx]

**Overall Comment: Minor Revisions**

**Dear respectful editor and reviewer,**

**We would like to appreciate your comments to improve the paper quality. We tried our best to address all the comments. Hope that the revised paper satisfies the publications’ requirements.**

**Sincerely,**

**Corresponding author**

**Response to Reviewer Comment**

**Reviewer #1:**

**Comment-** **Authors need to add a table of used symbols in the paper to make the paper read easier.**

**Response: Thank you for your valuable suggestion to include a table of used symbols in our paper. We recognize the importance of such a table in enhancing the readability and accessibility of our manuscript, especially for complex equations and technical discussions where various symbols are extensively used.**

**To address this, we added a comprehensive table of symbols in Appendix A of the paper, immediately following the conclusion section. This table lists each symbol used, along with its definition.**

**Line 627:**

**“*Appendix A: Table of Symbols***

*This appendix lists all symbols used throughout the paper along with their definitions to aid in the reader's understanding.*

| Symbol | Description |
| --- | --- |
| N | Number of distinct users |
| U | Set of users, $U=\left\{ u_{1}, u_{2},\ldots,u_{N} \right\}$ |
| M | Number of images |
| I | Set of images, $I=\{I_{1},I_{2},\ldots,I_{M}\}$, |
| $\boldsymbol{I}_{\boldsymbol{i}}$ | Subset of images liked by user I, $I_{i}=\{x_{1}^{i},x_{2}^{i},\ldots,x_{k}^{i}\}$ |
| S | State space |
| A | Action space |
| R | Reward |
| P | Transition probability |
| γ | Discount factor |
| $\boldsymbol{e}_{\boldsymbol{i}}$ | the feature vector representation of item $x^{i}$ |
| $\boldsymbol{score}_{\boldsymbol{i}}$ | Scoring function for image recommendation, ${score}_{i}=e_{i}^{T}a$ |
| $\boldsymbol{L}\left( \boldsymbol{\theta}^{\boldsymbol{Q}} \right)$ | Loss function for the critic network |
| $\boldsymbol{L}\left( \boldsymbol{\theta}^{\boldsymbol{\mu}} \right)$ | Loss function for the actor network |
| $\boldsymbol{Q}\left( \boldsymbol{s,a} \vert\boldsymbol{\theta}^{\boldsymbol{Q}} \right)$ | Q-value of state-action pair under critic network parameters |
| y | Target value for critic network update |
| $\boldsymbol{s}_{\boldsymbol{N}}$ | Next state of the user |
| $\boldsymbol{R}_{\boldsymbol{I}}$ | Recommended item at the current step |
| $\boldsymbol{w}_{\boldsymbol{ij}}$ | Weight in the self-attention mechanism |
| $\boldsymbol{x}_{\boldsymbol{j}}$ | Image previously liked by the user |
| $\boldsymbol{\beta}$ | Coefficient vector for recommendation memory |
| $\boldsymbol{score}_{\boldsymbol{i\beta}}$ | Score for image recommendation using memory vector |

”

**Comment-** **What specific improvements the authors consider regarding the methodology?**

**Response: To answer this comment, we have modified the introduction section as follows:**

**Line 51:** “*Our proposed method comprises two main parts with following innovations in each one. In the first part, we aim to extract a diverse set of features from images to capture the characteristics and preferences of the user. For this purpose, we introduce three novel components: emotion analysis, personality recognition, and style detection. These elements are designed to represent users more accurately and enhance the social image recommender system. In the second part, we introduce a new framework based on actor-critic RL, complemented by a state representation module, to establish a dynamic recommender system.*

*The main contributions of this study can be summarized as follows:*

*1) Proposing an image recommender system in a new deep RL framework. Unlike traditional methods that treat recommendation as a static process, our methodology acknowledges the dynamic nature of user preferences. By utilizing a deep RL framework, our system not only reacts to user feedback in real-time but also predicts future preferences. This approach improves the system's efficiency in making recommendations with limited data.*

*2) Introducing a new method for state representation. We applied the concept of self-attention, a core mechanism of the Transformer, to compute the new state in our system. This method focuses on measuring dependencies within a sequence's components, offering a more nuanced understanding of user preferences.*

*3) Introducing three components (style, Emotion, personality) that can be useful in images recommendation to create user's characteristics vector and investigate its effect on the recommended images on each user. These features are not commonly used in current systems but have shown to significantly impact the system's ability to make personalized recommendations, as demonstrated in our test results.*

*In summary, our methodology introduces significant improvements by adopting a dynamic perspective on user preferences, integrating novel features for personalization, and leveraging the latest advances in deep learning and reinforcement learning. These enhancements not only address the challenges faced by existing systems but also pave the way for future research in the field.”*

**Comment-** **Language of the paper is too poor. Need a rigorous look. In many places few words are repeatedly used in one sentence or paragraph. That degrades the readability of the paper. Rewrite abstract.**

**Response: The language of the manuscript was reviewed and grammatical errors were corrected.**

**Comment-** **What is your fitness function? What is the outcome of the proposed algorithm?**

**Response: we propose a new social image recommender system within the deep RL framework. For this purpose, the deep deterministic policy gradient (DDPG) algorithm has been employed to generate recommendations. DDPG is an actor-critic method that merges the strategies of Q-learning and policy gradients, consisting of two models: the Actor network and the Critic network.**

**In the context of the DDPG algorithm, the fitness function, often referred to as the reward function in reinforcement learning, is designed to evaluate the performance of the agent in its environment. The specific choice of a fitness function is crucial as it directly influences the agent's learning trajectory by dictating what behaviors are considered optimal. The fitness function used in our DDPG implementation is crafted to directly align with the objectives of our project, guiding the agent toward desirable behaviors, and is defined as follows:**

$\boldsymbol{Reward}\left( \boldsymbol{Image} \right)\boldsymbol{=}\left\{ \begin{matrix} \begin{aligned} \boldsymbol{1 If the user likes the image} \\ \boldsymbol{-1 If the user does not like the image} \end{aligned} \\ \end{matrix} \right.$	**(5)**

**If a recommended image is liked by the user, the system receives a positive reward; conversely, if the image is not liked, the system receives a negative reward.**

**In the article, there is a complete description of the used algorithm as follows:**

**Line 262:** “*In the Deep Deterministic Policy Gradient (DDPG) algorithm, both the actor and critic networks have their respective loss functions, which guide the learning process. These loss functions are central to the training and updating mechanisms. The critic is updated, with the objective of minimizing the difference between the predicted Q-values and the target Q-values. The loss function for the critic is typically the Mean Squared Error (MSE) between these two values:*

$L\left( \theta^{Q} \right)= \mathbb{E}_{(s,a,r,s^{'}\mathcal{)\sim D}}\left[ \left( Q\left( s,a | \theta^{Q} \right)-y \right)^{2} \right]$	*(2)*

*Where* $\theta^{Q}$ *are the parameters of the critic network, and* $y$ *is the target value, computed as* $y=r+\gamma Q^{'}(s^{'},\mu^{'}(s^{'}|\theta^{\mu^{'}})|\theta^{Q^{'}})$*. Here,* $\gamma$ *is the discount factor.* $Q^{'}(s^{'},\mu^{'}(s^{'}|\theta^{\mu^{'}})|\theta^{Q^{'}})$ *is the target Q-value for the next state, using the target critic network's parameters,* $\theta^{Q^{'}}$*, and the target actor network,* $\mu^{'}$*, for action selection.* $\mathcal{D}$ *represents a replay buffer containing tuples of (state, action, reward, next state).*

*The policy of the actor is updated using policy gradient methods. The objective is to maximize the Q-value, output by the critic, for the actions chosen by the actor. However, in practice, gradient ascent is performed on the expected Q-values predicted by the critic for the current policy. The loss function for the actor essentially becomes the negative of the expected Q-value:*

$L\left( \theta^{\mu} \right)= \mathbb{-E}_{s\sim\mathcal{D}}\left[ Q\left( s,\mu(s | \theta^{\mu} \right)|\theta^{Q}) \right]$	*(3)*

*Where* $\theta^{\mu}$ *are the parameters of the actor network.* $\mu(s,\theta^{\mu})$ *is the current policy's action given state* $s$*. The expectation is approximated by sampling from the replay buffer.*

*For each iteration (or batch of experiences), the critic's parameters* $\theta^{Q}$*are updated by minimizing the critic loss. This typically involves calculating gradients of* $L(\theta^{Q})$ *with respect to* $\theta^{Q}$ *and then performing a step of gradient descent. The actor's parameters* $\theta^{\mu}$ *are updated by performing gradient ascent on the expected Q-values. The gradients required for the update are derived from the critic's Q-value output with respect to the actor's parameters. This is where the dependency of the actor on the critic's assessment comes into play, guiding the actor to choose actions that the critic values more. These updates are typically applied in a loop, where a batch of experiences is sampled from the replay buffer, the critic is updated, and then the actor is updated. Target networks for both the actor and the critic are also maintained, with their weights being slowly tracked to the main networks' weights to stabilize learning. This process repeats for many episodes and steps, gradually improving both the actor's policy and the critic's value estimation.*

*Given the vast number of images available for recommendation, we propose utilizing a memory of the user's recommendation history to aid the recommendation process with the help of the Action vector. This memory records all images recommended to the user, including those they liked and those they did not. We employ the Lasso algorithm to derive a vector of coefficients from the stored information. We then calculate the dot product between this coefficient vector (*$\beta$*) and the candidate images to select a list that represents the most suitable recommendations.*

${score}_{i\beta}=x_{j}\beta$	*(4)*

*After calculating the* ${score}_{j\beta}$ *for all* $x_{j}$*, we consider those with higher scores as better candidates for recommendation. We then compute the dot product of the list of selected images with the action vector obtained from the actor network to create recommendations, as outlined in Eq. (1).The reward function is defined in Eq. (5). If a recommended image is liked by the user, the system receives a positive reward; conversely, if the image is not liked, the system receives a negative reward.*

$Reward\left( Image \right)= \left\{ \begin{matrix} \begin{aligned} 1 If the user likes the image \\ -1 If the user does not like the image \end{aligned} \\ \end{matrix} \right.$	(5)

”

**As for the outcomes of applying DDPG to our image recommendation problem, we have observed improvements the result of images recommendation in comparisons with other methods (the results reported in figures 6 to 10). These results show the effectiveness of DDPG in learning complex policies in continuous action spaces.**

**Indeed, Due to encounter with the huge volume and variety of information, we need a system that can automatically identify and cater to the user's interests. The remarkable point about previous methods is that they often view the recommendation process as static, assuming that a user's underlying preferences remain unchanged. In reality, personalizing image recommendations according to individual user preferences is a complex task, as their preferences evolve over time. This evolution necessitates dynamic interaction between the system and the user, indicating the need for a dynamic recommendation process.**

**Comment-** **Conduct a more thorough comparison with state-of-the-art algorithms, such as Hybrid Feature Selection Techniques Utilizing Soft Computing Methods for Cancer Data, Gene selection with Game Shapley Harris hawks optimizer for cancer classification, CO‐WOA: Novel Optimization Approach for Deep Learning Classification of Fish Image.**

**Response: Thank you for your comment and the suggestion to compare our method with the papers mentioned: "Hybrid Feature Selection Techniques Utilizing Soft Computing Methods for Cancer Data," "Gene selection with Game Shapley Harris hawks optimizer for cancer classification," and "CO‐WOA: Novel Optimization Approach for Deep Learning Classification of Fish Image." We appreciate the opportunity to discuss the relevance of these algorithms to our work further.**

**Below is an explanation of the work done in each of these valuable articles.**

**Hybrid Feature Selection Techniques Utilizing Soft Computing Methods for Cancer Data:** The article presents a hybrid machine learning framework that leverages soft computing techniques for feature selection, specifically targeting the challenge of processing vast microarray data in cancer diagnosis. It uses a two-stage process: first, extracting genes using higher-order Independent Component Analysis (ICA), then selecting the best genes via the Genetic Bee Colony (GBC) optimization technique. This approach is compared against other optimization methods (PSO, ABC, GA) and evaluated using Naïve Bayes and Support Vector Machine classifiers across five benchmark microarray datasets.

**Gene selection with Game Shapley Harris hawks optimizer for cancer classification:** The article presents a novel two-phase feature (gene) selection approach for cancer classification using high-dimensional gene expression data. Initially, it utilizes the kernel Shapley value (kSV) for extracting significant features based on a cooperative game-theoretic method. In the second phase, it employs the Harris hawks optimizer (HHO) algorithm for optimizing the feature subset selected by kSV. The effectiveness of this method is demonstrated through extensive experiments on eight benchmark datasets, comparing it with state-of-the-art techniques and employing SVM, Naive Bayes, and K-nearest neighbors classifiers.

**CO‐WOA: Novel Optimization Approach for Deep Learning Classification of Fish Image:** The article introduces a method for classifying fish species using an advanced computer vision technique called the Chaotic Oppositional Based Whale Optimization Algorithm (CO-WOA). This approach combines cutting-edge computer vision, the whale optimization algorithm, and data mining techniques to achieve high accuracy rates. The method outperforms conventional models like CNN and VGG-19 by achieving a 100% accuracy rate in tests, demonstrating its superiority over existing image processing models in the challenging task of fish image classification.

**Given the summaries we've provided for each article, each work introduces innovative methods within their respective domains.**

**We have thoroughly reviewed the suggested articles and acknowledge its contributions within their respective domains. However, after careful consideration, we believe that a direct comparison between our work and the suggested articles may not be appropriate or informative for the following reasons:**

**Divergent Domains: Our research focuses on the development of a dynamic image recommender system for social networks, which adapts to changing user preferences by employing a deep reinforcement learning framework. This framework leverages unique features such as emotion, style, and personality to enhance the personalization of image recommendations.**

**Relevance to the Audience: Our manuscript aims to address the challenges faced by the social image recommendation community, particularly the need for dynamic strategies that adapt to user preferences over time. The insights and findings presented in our work are thus most relevant to researchers and practitioners in the fields of recommender systems, social networks, and personalized content delivery. The suggested articles, while valuable, addresses a different audience and set of research questions.**

**Given these considerations, we propose to maintain the focus of our comparison on works that are more closely aligned with the objectives, methodologies, and audience of our research. We believe this will provide the most value to readers and more accurately situate our contributions within the existing body of knowledge on dynamic recommender systems and personalized content delivery.**

**However, we acknowledge the importance of interdisciplinary insights and will reference the suggested articles to highlight the diversity of approaches across different domains. This gesture aims to encourage cross-pollination of ideas where applicable but emphasizes that a direct comparison might not accurately reflect the contributions and innovations of our work in the context of social image recommendation.**

**The articles “Hybrid Feature Selection Techniques Utilizing Soft Computing Methods for Cancer Data”, “CO‐WOA: Novel Optimization Approach for Deep Learning Classification of Fish Image”, and “Gene selection with Game Shapley Harris hawks optimizer for cancer classification” have been added as references [6], [7], and [8] respectively.**

**Line 25:** “*Prior studies have explored handcrafted features [1, 2], as well as deep learning models [3-5] for image representation and recommendation. Feature selection and the use of deep learning models have been prominent in many studies [6-8]. In recommender systems, various similarity measures [9] and classification methods [10] have been employed.*”

**Comment-** **Provide a balanced discussion on the advantages and drawbacks of the proposed work.**

**Response: To response to this comment, we have added description to the Implementation Results and Analysis section as follows:**

**Line 575:** “*In the following, we explore the advantages and drawbacks of our proposed framework. User-Centric Design stands out as a significant advantage. Our system comprehensive approach, incorporating user preferences in terms of emotion, style, and personality, represents a significant advancement in personalization. This level of customization ensures that recommendations are more aligned with individual users' tastes, potentially enhancing user satisfaction and engagement. Additionally, Interactive Learning is another advantage. The system's interactive nature, which adapts and updates based on user feedback, is a major strength. This dynamic learning process enables the system to become more accurate over time, tailoring its recommendations more effectively to each user's evolving preferences. Furthermore, the ability to initiate learning without requiring a large dataset from the onset is a noteworthy advantage. This feature makes the system particularly valuable for new users or in applications where historical data is limited, ensuring a seamless and engaging user experience from the start. Continuous Improvement is yet another advantage. The proposed method's capacity to interact with the user from the beginning and gradually improve recommendations demonstrates its potential for real-world applications, where user preferences can vary widely and change over time.*

*In contrast, the significant training time required due to the model depth and complexity, especially when compared to other methods, could be seen as a drawback. Additionally, the space complexity associated with maintaining dual neural networks, the experience replay buffer, and a memory of recommendation history might pose challenges. While the advanced features and interactive approach are beneficial for personalization, they demand substantial computational resources. Although the system offers a high degree of personalization, there's a trade-off between the model's complexity and its practicality.*

*In conclusion, our proposed dynamic image recommender system presents a novel and user-centric approach to personalization, leveraging deep reinforcement learning and a broad spectrum of user preferences. While the advantages highlight its potential for highly tailored recommendations and adaptability, the drawbacks show the challenges related to training time, space complexity, and computational demands. Balancing these factors is crucial for maximizing the system's real-world applicability and user satisfaction.*”
